# Elevated gonadotropin levels are associated with increased biomarker risk of Alzheimer’s disease in midlife women

**DOI:** 10.3389/frdem.2023.1303256

**Published:** 2023-11-23

**Authors:** Matilde Nerattini, Federica Rubino, Steven Jett, Caroline Andy, Camila Boneu, Camila Zarate, Caroline Carlton, Susan Loeb-Zeitlin, Yelena Havryliuk, Silky Pahlajani, Schantel Williams, Valentina Berti, Paul Christos, Matthew Fink, Jonathan P. Dyke, Roberta Diaz Brinton, Lisa Mosconi

**Affiliations:** 1 Department of Neurology, Weill Cornell Medicine, New York, NY, United States; 2 Department of Experimental and Clinical Biomedical Sciences, Nuclear Medicine Unit, University of Florence, Florence, Italy; 3 Department of Population Health Sciences, Weill Cornell Medicine, New York, NY, United States; 4 Department of Obstetrics and Gynecology, Weill Cornell Medicine, New York, NY, United States; 5 Department of Radiology, Weill Cornell Medicine, New York, NY, United States; 6 Department of Neurology and Pharmacology, University of Arizona, Tucson, AZ, United States

**Keywords:** gonadotropin (FSH and LH), Alzheimer’s disease, neuroimaging, menopause, women, positron emission tomography (PET), Magnetic Resonance Imaging (MRI)

## Abstract

**Introduction:**

In preclinical studies, menopausal elevations in pituitary gonadotropins, follicle-stimulating hormone (FSH) and luteinizing hormone (LH), trigger Alzheimer’s disease (AD) pathology and synaptic loss in female animals. Herein, we took a translational approach to test whether gonadotropin elevations are linked to AD pathophysiology in women.

**Methods:**

We examined 191 women ages 40–65 years, carrying risk factors for late-onset AD, including 45 premenopausal, 67 perimenopausal, and 79 postmenopausal participants with clinical, laboratory, cognitive exams, and volumetric MRI scans. Half of the cohort completed ^11^C-Pittsburgh Compound B (PiB) amyloid-β (Aβ) PET scans. Associations between serum FSH, LH and biomarkers were examined using voxel-based analysis, overall and stratified by menopause status. Associations with region-of-interest (ROI) hippocampal volume, plasma estradiol levels, APOE-4 status, and cognition were assessed in sensitivity analyses.

**Results:**

FSH levels were positively associated with Aβ load in frontal cortex (multivariable adjusted *P*≤0.05, corrected for family wise type error, FWE), an effect that was driven by the postmenopausal group (multivariable adjusted *P*_*FWE*_ ≤ 0.044). LH levels were also associated with Aβ load in frontal cortex, which did not survive multivariable adjustment. FSH and LH were negatively associated with gray matter volume (GMV) in frontal cortex, overall and in each menopausal group (multivariable adjusted *P*_*FWE*_ ≤ 0.040), and FSH was marginally associated with ROI hippocampal volume (multivariable adjusted *P* = 0.058). Associations were independent of age, clinical confounders, menopause type, hormone therapy status, history of depression, APOE-4 status, and regional effects of estradiol. There were no significant associations with cognitive scores.

**Discussion:**

Increasing serum gonadotropin levels, especially FSH, are associated with higher Aβ load and lower GMV in some AD-vulnerable regions of midlife women at risk for AD. These findings are consistent with preclinical work and provide exploratory hormonal targets for precision medicine strategies for AD risk reduction.

## Introduction

Accumulating evidence from preclinical and translational studies implicates the loss of neuroprotective effects of sex steroid hormones following menopause as a female-specific risk factor for late-onset Alzheimer’s disease (AD) (Brinton et al., 2015; Rahman et al., 2019; Jett et al., 2022b). Menopause exerts its actions on AD risk via alterations of multiple neurobiological mechanisms that can span decades (Brinton et al., 2015), thus proximate to the beginning of the ~20 year prodromal phase of the disease (Sperling et al., 2013).

In mechanistic analyses, the menopause transition impacts cerebral bioenergetic aging processes, triggering amyloid-β (Aβ) plaque aggregation, mitochondrial compromise, and synaptic dysfunction (Brinton et al., 2015). In translational studies, women of menopausal age exhibit higher Aβ deposition (Mosconi et al., 2017, 2018b, 2021; Rahman et al., 2020), lower brain glucose metabolism and gray matter volume (GMV) (Mosconi et al., 2017, 2018b, 2021; Kim et al., 2018; Rahman et al., 2020; Schelbaum et al., 2021), as well as higher white matter hyperintensity (WMH) burden (Lohner et al., 2022) as compared to premenopausal women and/or age-controlled men. Postmenopausal women also exhibit higher brain tau levels than men with comparable cortical Aβ burden (Buckley et al., 2022). Surgical menopause has been associated with lower medial temporal lobe GMV (Zeydan et al., 2019), higher WMH load (Lohner et al., 2022), and greater neuropathological burden (Bove et al., 2014; Coughlan et al., 2023) than spontaneous menopause.

While estrogen declines, especially 17β-estradiol, are considered the primary trigger for menopause-related AD risk (Brinton et al., 2015; Jett et al., 2022b), clinical research has provided contrasting evidence for associations between estradiol and AD risk in women (Casadesus et al., 2004; Jett et al., 2022a). Additionally, while observational studies of menopause estrogen therapy report generally positive outcomes, randomized clinical trials have not shown consistent AD risk reduction effects (Maki, 2008; Jett et al., 2022a,b).

While estrogen continues to be investigated, these disparities prompted examination of other hypothalamic-pituitary-gonadal axis (HPG) hormones, chiefly follicle-stimulating hormone (FSH) and luteinizing hormone (LH). FSH and LH stimulate ovulation and estrogen production in the ovary and are regulated by negative feedback from estradiol levels. In contrast to estradiol, which exhibits wide fluctuations before reaching persistently low levels postmenopause, gonadotropin levels increase steadily starting in perimenopause (Monteleone et al., 2018), with over 10 and 3-fold increases, respectively (Chakravarti et al., 1976). Novel research has established a connection between these pituitary gonadotropins and AD risk (Bowen et al., 2000; Casadesus et al., 2004, 2006a; Xiong et al., 2022). FSH and LH elevations increase amyloidogenic processing of amyloid precursor protein (APP) in cell cultures, while pharmacological suppression of the gonadotropins reduces Aβ plaque formation (Bowen et al., 2000; Casadesus et al., 2004, 2006a; Xiong et al., 2022). In transgenic mouse models of AD, FSH elevations accelerate both Aβ and tau deposition, whereas FSH blockade prevents emergence of AD pathology (Xiong et al., 2022). Both overexpression of LH and its exogenous administration negatively affect cerebral Aβ production and hippocampus-dependent learning (Casadesus et al., 2007; Wahjoepramono et al., 2011), while the ablation of LH attenuates these effects (Casadesus et al., 2006b). It remains unknown whether gonadotropin elevations are linked to AD pathophysiology in women.

Herein, we took a translational approach to examine whether increasing FSH and LH from premenopausal to postmenopausal levels were associated with biomarker evidence of AD risk, as reflected in higher Aβ deposition and lower GMV (a marker of neuronal loss) in AD-vulnerable regions of midlife women at risk for AD.

## Methods

### Participants and data

This is a natural history, non-interventional study of cognitively normal women ages 40–65 years carrying risk factors for late-onset AD such as a family history and/or APOE-4 genotype. Participants were enrolled at the Weill Cornell Medicine (WCM) Alzheimer’s Prevention Program between 2018 and 2022. Our inclusion and exclusion criteria have been previously described (Mosconi et al., 2017, 2018b, 2021; Rahman et al., 2020; Schelbaum et al., 2021). Briefly, all participants had Montreal Cognitive Assessment (MoCA) score ≥26 and normal cognitive test performance by age and education. Exclusion criteria were medical conditions that may affect brain structure or function (e.g., stroke, any neurodegenerative diseases, major psychiatric disorders, hydrocephalus, demyelinating disorders, intracranial mass, and infarcts on Magnetic Resonance, MRI), use of psychoactive medications, and contraindications to MRI or PET. Presence of depression was assessed via the Hamilton test and/or the Patient-Reported Outcomes Measurement Information System (PROMIS) Depression measure. All participants received clinical, laboratory, cognitive exams and volumetric MRI within 6 months of each other. Half of the participants also received ^11^C-Pittsburgh Compound B (PiB) PET to measure Aβ load. The patients’ sex was determined by self-report. APOE-4 genotype was determined using standard qPCR procedures (Mosconi et al., 2017, 2018a,b, 2021; Rahman et al., 2020; Schelbaum et al., 2021). Participants carrying one or two copies of the APOE-4 allele were grouped together as APOE-4 carriers, and compared to non-carriers. A family history of late-onset AD was elicited using standardized questionnaires.

### Standard protocol approvals, registrations, and patient consents

All experimental protocols were approved by the WCM Institutional Review Board. Written informed consent was obtained from all participants.

### Cognitive measures

Participants underwent a cognitive testing battery with known sensitivity to estrogen levels (Maki and Henderson, 2016; Mosconi et al., 2018b, 2021): [delayed recall of Rey Auditory Verbal Learning Test (RAVLT) and Wechsler Memory Scale logical memory], executive function [Trail Making Test, FAS], and language [object naming, animal naming]. A composite memory score was obtained by z-scoring each delayed recall memory test and averaging across measures. A global cognition score was obtained by z-scoring all tests by domain, and averaging within and across domains.

### Hormonal panel

All participants received a blood draw by venipuncture after an overnight fast. Samples were shipped overnight to CLIA-certified Boston Heart Diagnostics [Framingham, MA] and analyzed on a Roche Cobas e801 analytical unit for immunoassay tests using Electrochemiluminescence technology (ECL) [Roche Diagnostics; Basel, Switzerland]. FSH and LH were assessed through electrochemiluminescence sandwich immunoassay (ELISA) with a measuring range of 0.3–200 mIU/mL for both hormones. Estradiol (E2) was assessed through competitive immunoassay with a measuring range of 18.4–11,010 pmol/L (5–3,000 pg/mL).

### Menopause assessments

We focused on women at different menopausal stages (pre-menopause, peri-menopause, and post-menopause) as a natural experiment of increasing follicle stimulating hormone (FSH) levels across the menopause transition. As such, participants were not randomly assigned to groups. Determination of menopausal status was based on the Stages of Reproductive Aging Workshop (STRAW) criteria (Harlow et al., 2012) with hormone assessments as supportive criteria (Schelbaum et al., 2021). Participants were classified as premenopausal (regular cycler), perimenopausal (irregular cyclers with interval of amenorrhea ≥ 60 days or ≥2 skipped cycles) and postmenopausal (no cycle for ≥ 12 months) (Schelbaum et al., 2021). A history of hysterectomy and/or oophorectomy before menopause was assessed through review of surgical history. Semi-standardized questionnaires were used to obtain information on history of menopause hormone therapy (MHT) and oral contraceptive (OCP) usage. This information was used to classify participants as current vs. past or never users of MHT or OCP (Schelbaum et al., 2021).

### Image acquisition and analysis

Brain scans were acquired following standardized procedures (Mosconi et al., 2017, 2018b, 2021; Rahman et al., 2020; Schelbaum et al., 2021). Participants received a 3D volumetric T_1_-weighted MRI scan on a 3.0 T GE MR 750 Discovery scanner (General Electric, Waukesha, WI) [Brain Volume Imaging (BRAVO); 1 × 1 × 1 mm resolution, 8.2 ms repetition time (TR), 3.2 ms echo time (TE), 12° flip angle, 25.6 cm field of view (FOV), 256 × 256 matrix with ARC acceleration] using a 32-channel head coil. The ^11^C-PIB PET scan was acquired using a Siemens BioGraph mCT 64-slice PET/CT operating in 3D mode [70 cm transverse FOV, 16.2 cm axial FOV]. Summed PET images were obtained 60–90 min post-injection of 15 mCi of ^11^C-PiB and corrected for attenuation, scatter and decay.

Image analysis was performed using a fully automated image processing pipeline (Mosconi et al., 2017, 2018b, 2021; Rahman et al., 2020; Schelbaum et al., 2021). MRI and PET scans were realigned using the Normalized Mutual Information routine of Statistical Parametric Mapping (SPM12) (Acton and Friston, 1998) implemented in Matlab R2018a (MathWorks; Natick, MA). Each PET scan was coregistered to the corresponding T1 BRAVO. The coregistered images were processed using SPM12. First, MRI scans were spatially normalized to the template tissue probabilistic map (TPM) image in SPM12, conforming to the Montreal Neurological Institute (MNI) space, and processed for voxel-based morphometry (VBM) (Ashburner and Friston, 2005) including image segmentation, Jacobian modulation, high-dimensional warping (DARTEL) of the segments, and application of an 8 mm full-width at half maximum (FWHM) smoothing kernel (Ashburner and Friston, 2005). Gray matter (GM) segments were retained for statistical analysis. Secondly, the MRI-coregistered PET scans were spatially normalized to the TPM image using the MRI subject-specific transformation matrices using the MRI as the anchor, and smoothed using an 8-mm FWHM filter. SPM12 was also used to obtain total intracranial volume (TIV) as the sum of gray, white and cerebrospinal fluid volume for each subject (Ashburner and Friston, 2005). Cerebellar gray matter PiB uptake was extracted using the automated anatomical labeling (AAL3) atlas (Tzourio-Mazoyer et al., 2002) and WFU PickAtlas 2.4 (Maldjian et al., 2003). GMV measures were adjusted by TIV. PiB measures were normalized to cerebellar gray matter uptake to obtain standardized uptake value ratios (SUVR).

### Covariates

Cognitive analyses were adjusted by age and education (years). Biomarker analyses were adjusted by age, menopause status, history of depression and modality-specific confounders (MRI TIV; cerebellar PiB uptake). For exposures showing significant associations with outcome measures, we further examined OCP status (user vs. non-user), MHT status (user vs. non-user) and menopause type (history of hysterectomy and/or oophorectomy vs. spontaneous menopause). APOE-4 status (carrier vs. non-carrier) was examined as a covariate and for interactions with hormone-biomarkers associations.

### Statistical analysis

Analyses were performed in R v.4.2.0, SPSS v.28, and SPM12.

Clinical measures were examined using general linear models or chi-squared tests as appropriate. Cohort characteristics are described using mean (standard deviation) and *n*, percentage (%), stratified by exposure group. As the hormone measures did not follow a Gaussian distribution, we used the automated bestNormalize R package to identify the optimal normalization transformation for each measure. The Ordered Quantile (ORQ) normalization transformation was selected as it maximized the statistics. Hippocampal ROI data and TIV obtained with FreeSurfer also required normalization. Following the transformations, all variables passed the Shapiro Wilks test for normality and their histograms displayed normal distributions.

We conducted several analyses to address the independent effects of FSH and LH levels from age and menopause status according to published methods (Becker et al., 2005; Rahman et al., 2020): (a) we used box plots and frequency diagrams to confirm that we had sufficient age overlap among women of different menopause statuses, which enabled us to examine the effects of FSH and LH separately from additional effects of menopause; (b) we used variance inflation factor (VIF) (Kutner et al., 2004) as a diagnostic tool to test for multicollinearity between exposures and age, using SPSS v.28. All VIF values were well below the critical threshold of 5, ranging from 1.55 to 1.78, indicating lack of significant collinearity according to commonly accepted guidelines (Kutner et al., 2004); (c) we included age and menopause status as covariates; (d) we conducted a stringent voxel-based subtraction analysis (Acton and Friston, 1998) to test for unique contributions of the hormone measures to biomarker outcomes independent of regional contributions of age and menopause status (see below); and (e) we tested for associations between FSH, LH and biomarker outcomes within each postmenopausal and perimenopausal groups, also adjusting by age.

### FSH and LH associations with brain biomarkers

We used multivariate linear regressions with *post-hoc t*-contrasts to test for voxel-based associations between exposures (FSH or LH) and biomarker outcomes (Aβ load and GMV), adjusting by covariates. Statistical maps were obtained at *P* < 0.05, cluster-level corrected for Family-Wise Type Error (FWE) within a binary masking image consisting of *a priori* defined regions with known vulnerability to AD (AD_MASK_), including frontal, parietal, temporal cortex, cingulate gyrus and precuneus, thalamus and medial temporal lobes (Jack et al., 2013). In analysis of PiB data, medial temporal regions were excluded from the mask, due to the relative lack of measurable Aβ plaque burden (Chen et al., 2021). AD_MASK_ was set as an explicit (inclusive) mask to conservatively restrict analysis to regions within the mask (Acton and Friston, 1998). Cluster extent was set at ≥16 voxels. Anatomical location of regions reaching significance was described using Talairach coordinates after conversion from MNI space.

In the interest of being maximally conservative, in addition to examining age and menopause status as confounders, we conducted a stringent subtraction analysis (Acton and Friston, 1998) in the framework of SPM12 to determine whether FSH and LH had entirely independent associations with Aβ load and GMV from age and menopause status; e.g., by testing for associations between hormones and biomarkers after *excluding* all regional contributions of age and menopause status. This was accomplished as follows:

- We used regression models to identify voxels with statisticallysignificant associations between each hypothesized confounder (age and menopause status) and biomarker outcome at *P* < 0.05, cluster-level FWE corrected within the AD_MASK_. Age had positive associations with Aβ load in precuneus, frontal and parietal cortices; and negative associations with GMV in frontal, parietal, cingulate, temporal cortices, and thalamus (*P*_FWE_ < 0.05; Supplementary Tables 1, 2). Menopausal status had positive associations with Aβ load in left superior frontal gyrus, and negative associations with GMV in right cingulate cortex and parahippocampal gyrus (*P*_FWE_ < 0.05; Supplementary Tables 1, 2).
- The above clusters were saved as binary masks (Age_MASK_, Menopause_MASK_) and excluded from subsequent analysis of hormone-biomarker associations. This was done via the exclusive masking option included at the t-map generation step in SPM12 (Acton and Friston, 1998). SPM12 was then employed to evaluate the independent associations between each exposure (FSH or LH) and the outcomes in the remaining voxels within the AD_MASK_, at *P* ^<^ 0.05_FWE_.

Biomarker measures were extracted from peak clusters as volumes-of-interest (VOI) using MarsBar 0.45 (https://marsbar-toolbox.github.io/download.html). Regression models were trained to test for associations between FSH, LH and each biomarker outcome at *P* < 0.05 across participants and within menopausal groups. Estimates and standard errors (SE) are presented for the overall study sample as well as for postmenopausal and perimenopausal statuses. The stratified analysis was performed to investigate hypothesized differences in the strength of the correlations by menopause stage and to mitigate the effects of its confounding on the overall correlations. Analyses were repeated after restricting analysis to MHT non-users.

### Sensitivity analysis

#### Hippocampal volume

There is evidence that voxel-based analysis is effective for larger cortical regions but may underestimate effects in medial temporal lobe regions such as hippocampus (Mosconi et al., 2005), a region with higher anatomical specificity for cognitive aging and AD (Jack et al., 2013). Therefore, we performed a sensitivity analysis to test for associations between gonadotropin levels and hippocampal volume as an *a priori* selected region, using a region-of-interest (ROI) approach. Volumetric MRI scans were processed using FreeSurfer 7.2 running under the Centos 7 Linux environment, with Desikan-Killiany Atlas-based ROIs (Desikan et al., 2006; Fischl, 2012). We focused on the hippocampus, a structure with known AD vulnerability, and calculated hippocampal volume as the sum left and right hippocampal ROIs. We also obtained total intracranial volume for normalization purposes. Regression models were trained to test for associations between exposures (FSH or LH) and hippocampal volume at *P* < 0.05, adjusting by the same covariates as above, using SPSS 28.0. Estimates and standard errors (SE) are presented for the overall study sample as well as for postmenopausal and perimenopausal statuses. Since the associations were only marginally significant, hippocampal volume was not included in further analyses.

#### APOE-4 interaction effects

Linear regression models were developed for each pair of biomarker outcomes (Aβ load and GMV extracted from the peak cluster for each modality) and exposures (FSH or LH) at *P* < 0.05. Each model contained each exposure, APOE-4 status, and their interaction as covariates, and additional covariates.

#### Effects of estradiol

The same procedures were used to test for associations between estradiol and imaging outcomes. We also conducted a subtraction analysis (Acton and Friston, 1998) to determine whether FSH and LH had independent associations from estradiol; e.g., by testing for associations between each exposure and the outcomes *excluding* all regional contributions of estradiol, as well as Age_MASK_ and Menopause_MASK_. Since estradiol did not have significant associations with PiB measures, this analysis was restricted to GMV.

#### Associations of FSH and LH with cognition

We used linear regressions to test for associations between FSH, LH and cognitive scores, overall and by menopause group, adjusting by confounders, at *P* < 0.05.

## Results

### Participants

We enrolled 205 participants for this study. Of these, 14 were excluded due to incidental findings on Magnetic Resonance Imaging (MRI) (*n* = 5 small vessel disease or lacunar infarctions, *n* = 2 meningiomas, *n* = 1 mild hydrocephalus, *n* = 1 demyelination), image artifacts (*n* = 2), or incomplete hormonal test results (*n* = 3). The remaining 191 participants were examined in this study, including 45 premenopausal, 67 perimenopausal, and 79 postmenopausal women.

Participant characteristics are shown in Table 1. Twenty percent of participants reported taking MHT, 5% OCP, and 10% had a history of hysterectomy and/or oophorectomy. Thirteen percent reported a history of mild depression.

### Associations between FSH, LH and AD biomarkers

#### Aβ load

**FIGURE 1:**
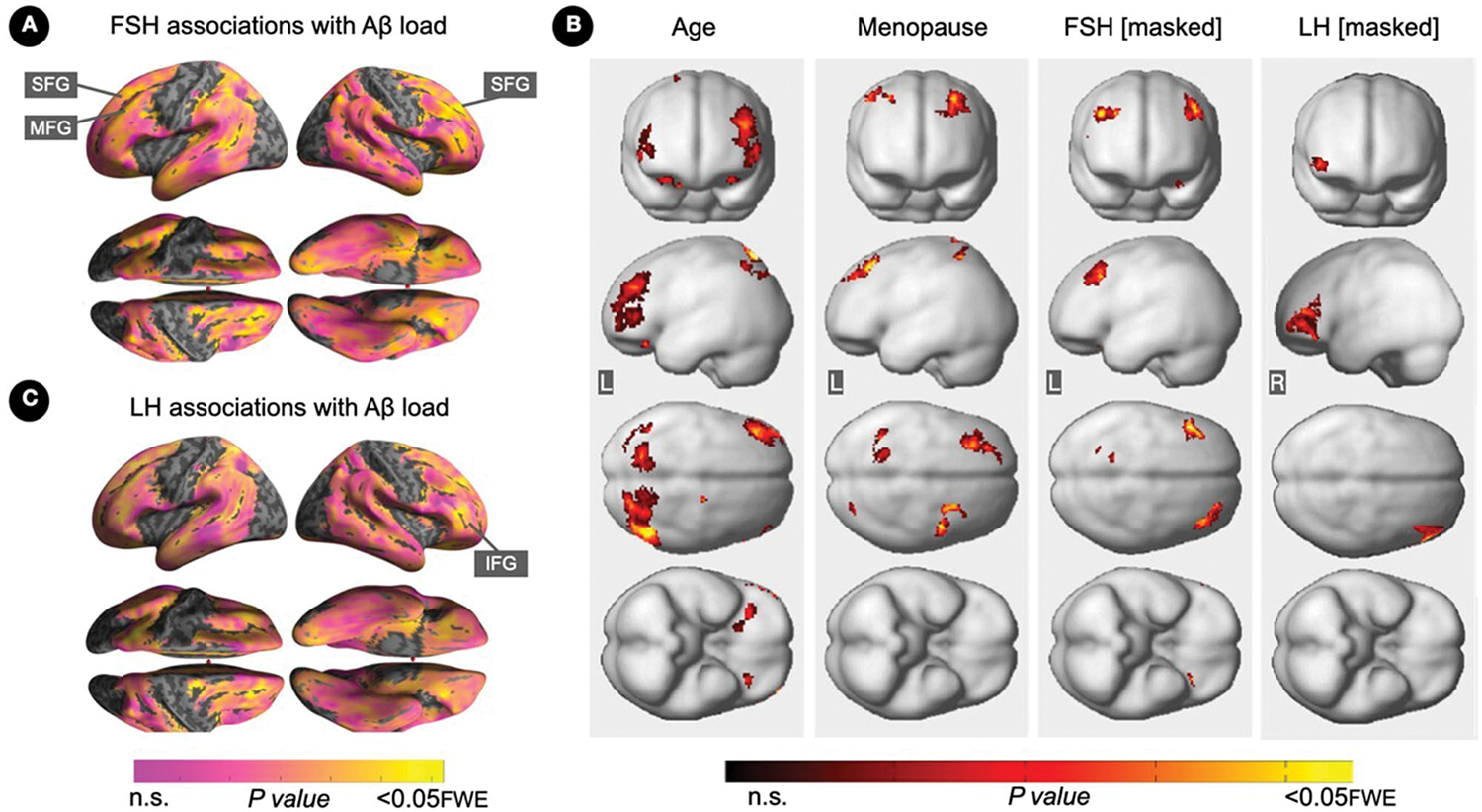
Associations of FSH and LH levels with Aβ load. **(A)** Surface maps of positive voxel-wise associations between FSH levels and ^11^C-PiB uptake, reflecting Aβ load. **(B)** Subtraction analysis testing for associations of FSH and LH with Aβ load independent of age and menopause status; e.g., *excluding* all voxel-wise regional contributions of age and menopause status using the explicit masking approach implemented in SPM12. From left to right, statistical parametric maps (SPMs) showing (i) regional associations between age and Aβ load; (ii) regional associations between menopause status and Aβ load; (iii) regional associations between FSH and Aβ load that survived the masking (e.g., masking out all voxels with age-Aβ load and menopause-Aβ load associations) and the multiple comparisons adjustment; (iv) regional associations between LH and Aβ load that survived the masking and the multiple comparisons adjustment. **(C)** Surface maps of voxel-wise associations between LH levels and Aβ load. Surface maps are generated within the prespecified search volume of AD-vulnerable regions and displayed on the lateral, superior and inferior views of an inflated, rendered MR image. In **(B)**, from top to bottom, SPMs are displayed on the anterior, lateral, superior and inferior views of a volume-rendered MR template image. Results are displayed using color-coded scales with corresponding *P*-values. FWE, family wise type errors; IFG, inferior frontal gyrus; L, left; MFG, middle frontal gyrus; R, right; SFG, superior frontal gyrus.

**FIGURE 2:**
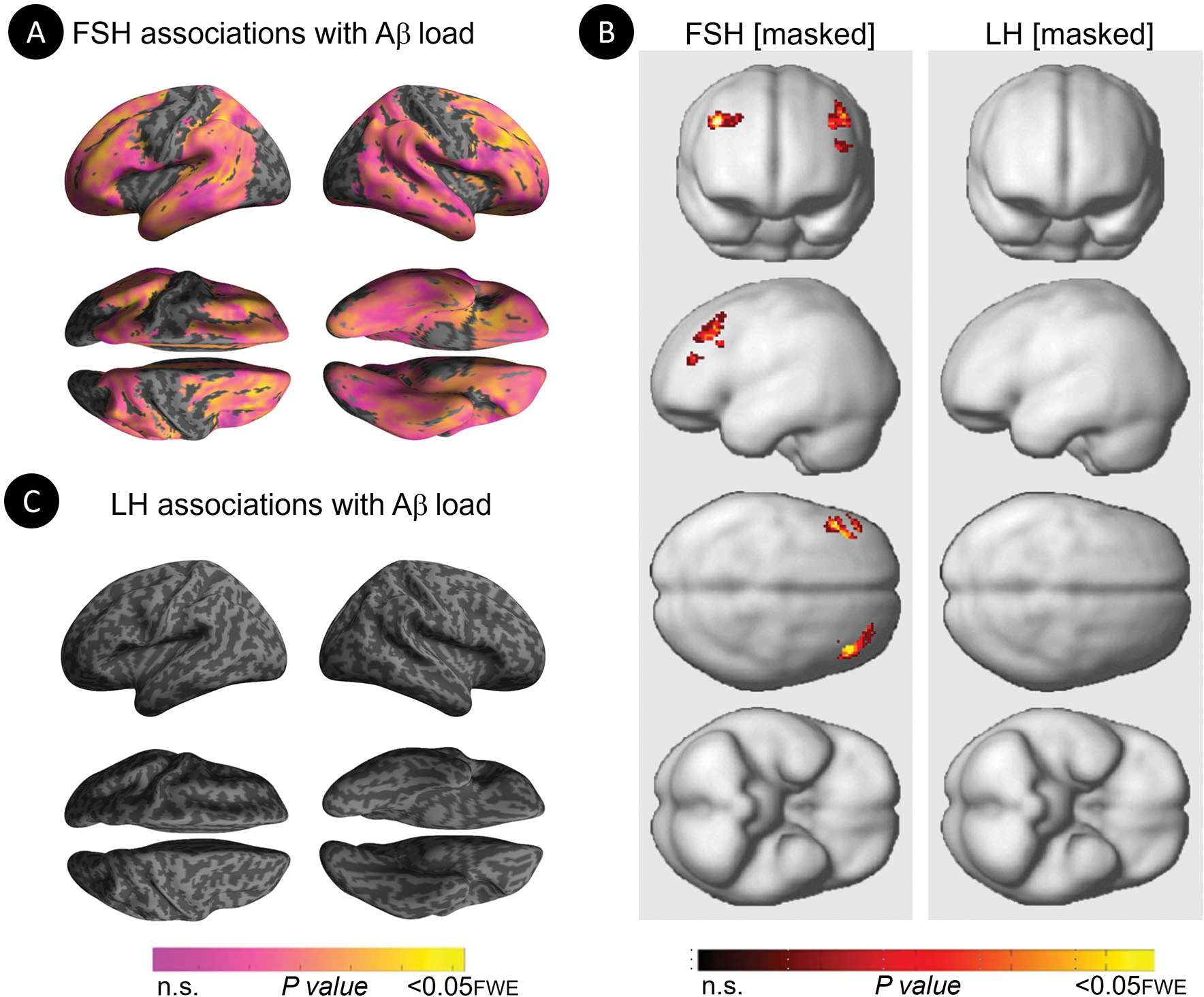
Associations of FSH and LH levels with Aβ load among MHT non-users. **(A)** Surface maps of positive voxel-wise associations between FSH levels and ^11^C-PiB uptake, reflecting Aβ load among MHT non-users. **(B)** Statistical parametric maps (SPMs) displaying positive voxel-wise associations between gonadotropins and Aβ load among MHT non-users, excluding all regional effects of age and menopause. From left to right, statistical parametric maps (SPMs) showing (i) regional associations between FSH and Aβ load that survived the masking (e.g., masking out all voxels with age-Aβ load and menopause-Aβ load associations among MHT non-users); (ii) regional associations between LH and Aβ load that survived the masking among MHT non-users. **(C)** Surface maps of voxel-wise associations between LH levels and Aβ load among MHT non-users. Surface maps are generated within the prespecified search volume of AD-vulnerable regions and displayed on the lateral, superior and inferior views of an inflated, rendered MR image. In **(B)**, from top to bottom, SPMs are displayed on the anterior, lateral, superior and inferior views of a volume-rendered MR template image. Results are displayed using color-coded scales with corresponding *P*-values. FWE, family wise type errors.

Increasing FSH levels were associated with higher Aβ load in middle frontal gyrus of the left hemisphere and superior frontal gyrus of both hemispheres, independent of age and menopause status (*P*_*FWE*_≤ 0.028; Table 2; Figures 1A, B). Associations between FSH and Aβ load remained significant in middle frontal gyrus after multivariable adjustment, (*P* = 0.044) and borderline significant in superior frontal gyrus (*P* = 0.065; Table 3). FSH-Aβ associations in middle frontal gyrus were driven by the postmenopausal group (multivariable adjusted *P* = 0.042) as no significant effects were found in the perimenopausal group (Table 3). After excluding MHT users, FSH levels remained associated with Aβ load in bilateral superior frontal gyrus, and middle frontal gyrus of the left hemisphere, independent of age and menopause status (*P*_*FWE*_ < 0.023; Table 4; Figures 2A, B).

Increasing LH levels were associated with higher Aβ load in inferior frontal gyrus of the right hemisphere, independent of age and menopause status (*P*_*FWE*_= 0.007; Table 2; Figures 1B, C). However, LH-Aβ associations did not survive multivariable adjustment, overall or by menopause status (Table 3), and did not reach significance among MHT non-users (Table 4; Figures 2B, C).

#### Gray matter volume

**FIGURE 3:**
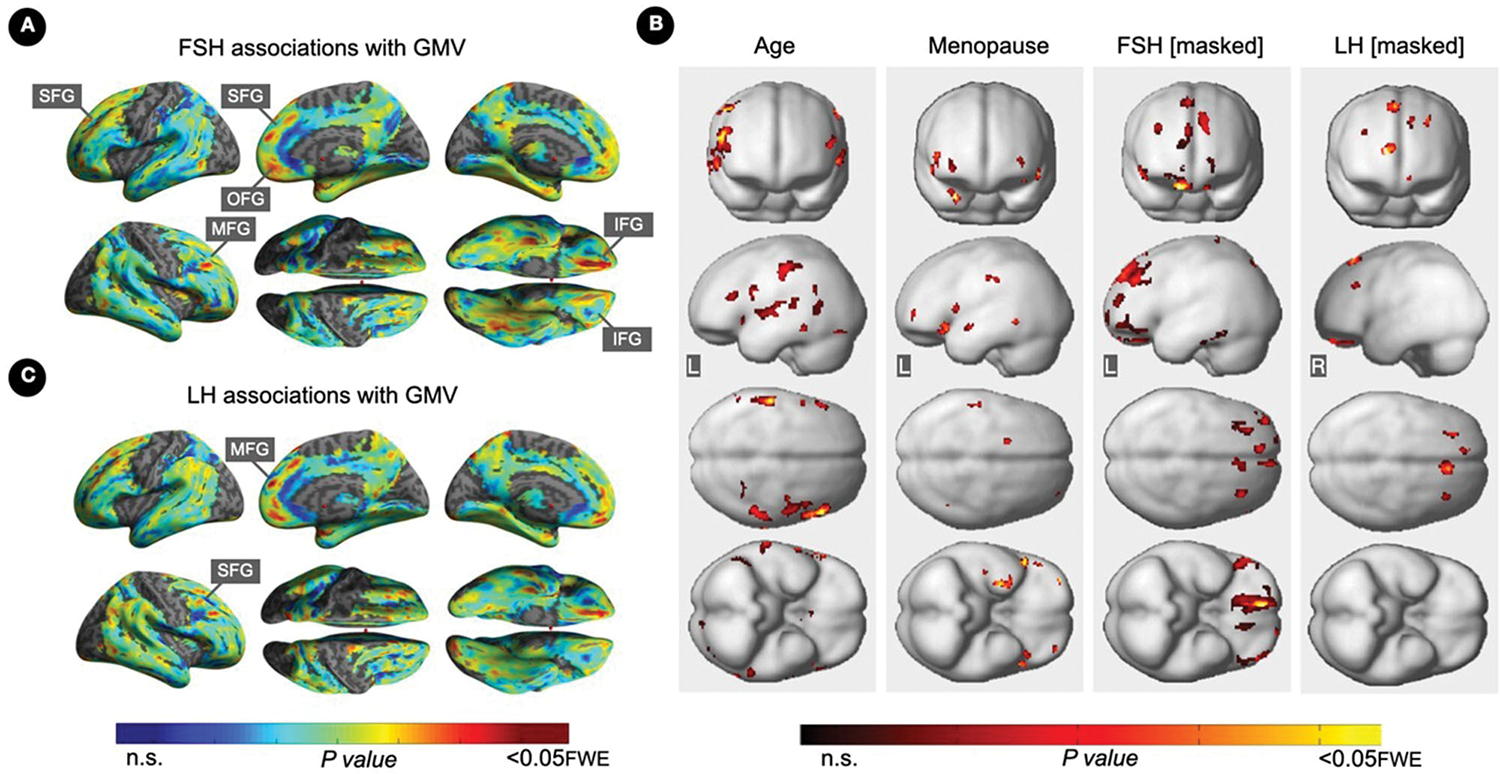
Associations of serum FSH and LH with gray matter volume. **(A)** Surface maps of negative voxel-wise associations between FSH levels and MRI gray matter volume (GMV). **(B)** Subtraction analysis testing for associations of FSH and LH with GMV independent of age and menopause status; e.g., *excluding* all voxel-wise regional contributions of age and menopause status. From left to right, statistical parametric maps (SPMs) showing: (i) regional associations between age and GMV; (ii) regional associations between menopause status and GMV; (iii) regional associations between FSH and GMV that survived the masking (e.g., masking out all voxels with age-GMV and menopause-GMV associations) and the multiple comparisons adjustment; (iv) regional associations between LH and GMV that survived the masking and the multiple comparisons adjustment. **(C)** Surface maps of negative voxel-wise associations between LH levels and GMV. Surface maps are generated within the prespecified search volume of AD-vulnerable regions including medial temporal lobe and displayed on the lateral, medial, superior and inferior views of an inflated, rendered MR image. In **(B)**, from top to bottom, SPMs are displayed on the anterior, lateral, superior and inferior views of a volume-rendered MR template image. Results are displayed using color-coded scales with corresponding *P*-values. FWE, family wise type errors; IFG, inferior frontal gyrus; L, left; MFG, middle frontal gyrus; OFG, orbitofrontal gyrus; R, right; SFG, superior frontal gyrus.

FSH levels were negatively associated with GMV in inferior, orbital and middle frontal gyri of the right hemisphere, and in superior frontal gyrus of both hemispheres, independent of age and menopause status (*P*_*FWE*_≤ 0.016; Table 5; Figures 3A, B). Associations between FSH and GMV were significant after multivariable adjustment (multivariable adjusted *P* ≤ 0.008) except for a trend in superior frontal gyrus (multivariable adjusted *P* = 0.059; Table 3). Both postmenopausal and perimenopausal groups exhibited associations between FSH and GMV in orbitofrontal gyrus (multivariable adjusted *P* ≤ 0.040), and the postmenopausal group exhibited additional associations in inferior frontal gyrus (multivariable adjusted *P* ≤ 0.004) (Table 3).

LH levels were negatively associated with GMV in medial and superior frontal gyri of the right hemisphere, independent of age and menopause status (*P*_*FWE*_≤ 0.003; Table 5; Figures 3B, C). Associations between LH and frontal GMV were significant after multivariable adjustment (*P* ≤ 0.001; Table 3). Both postmenopausal and perimenopausal groups exhibited LH-GMV associations in medial frontal gyrus, and the postmenopausal group also in superior frontal gyrus (multivariable adjusted *P* ≤ 0.008; Table 3).

**FIGURE 4:**
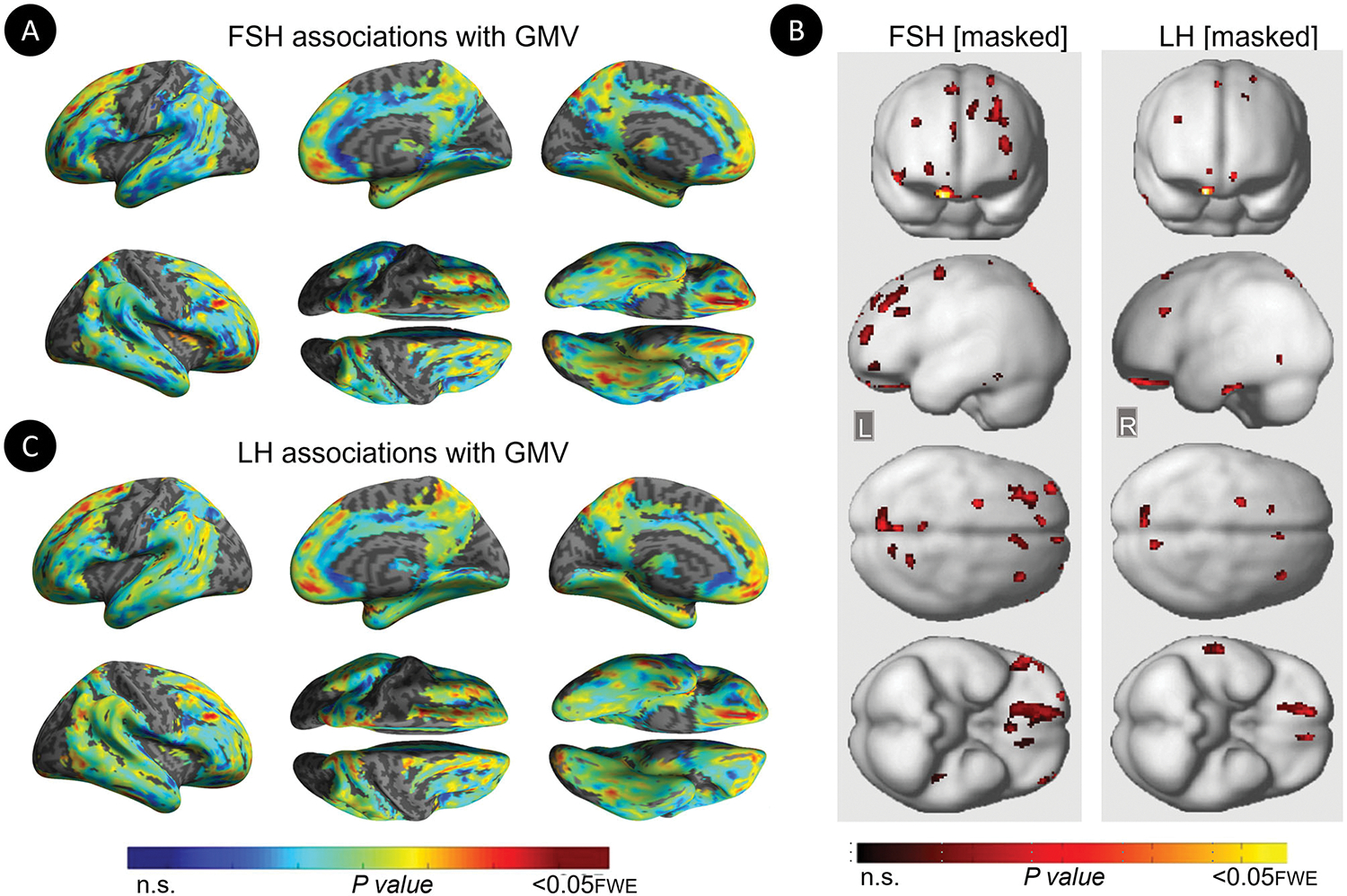
Associations of serum FSH and LH with gray matter volume among MHT non-users. **(A)** Surface maps of negative voxel-wise associations between FSH levels and MRI gray matter volume (GMV) among MHT non-users. **(B)** Statistical parametric maps (SPMs) displaying negative voxel-wise associations between gonadotropins and gray matter volume (GMV) among MHT non-users, excluding all regional effects of age and menopause. From left to right, statistical parametric maps (SPMs) showing: (i) regional associations between FSH and GMV that survived the masking (e.g., masking out all voxels with age-GMV and menopause-GMV associations among MHT non-users); (ii) regional associations between LH and GMV that survived the masking among MHT non-users. **(C)** Surface maps of negative voxel-wise associations between LH levels and GMV among MHT non-users. Surface maps are generated within the prespecified search volume of AD-vulnerable regions including medial temporal lobe and displayed on the lateral, medial, superior and inferior views of an inflated, rendered MR image. In **(B)**, from top to bottom, SPMs are displayed on the anterior, lateral, superior and inferior views of a volume-rendered MR template image. Results are displayed using color-coded scales with corresponding *P*-values. FWE, family wise type errors; L, left; R, righ.

Restricting analyses to MHT non-users left results largely unchanged (Table 6; Figure 4). FSH levels were negatively associated with GMV in middle frontal gyrus of both hemispheres, inferior and orbital gyri of the right hemisphere, and precuneus and fusiform gyrus of the left hemisphere (P_FWE_ ≤ 0.021). LH levels were negatively associated with GMV in middle and orbital gyri of the right hemisphere (P_FWE_ ≤ 0.007).

For the sake of completeness, we also analyzed GMV data at the whole-brain level. This exploratory analysis identified a few additional association clusters (Supplementary Table 3). For FSH-GM associations, this included right superior frontal gyrus and left insula (P_FWE_ ≤ 0.026), as well as rectal gyrus in analysis of MHT non-users (P_FWE_ = 0.037; Supplementary Table 3). For LH-GMV, additional clusters were noted in parahippocampal gyrus, amygdala and cerebellum of the right hemisphere of the entire cohort (P_FWE_ ≤ 0.011), and in right inferior temporal gyrus of MHT non-users (P_FWE_ = 0.006; Supplementary Table 3).

### Sensitivity analysis

#### Hippocampal volume

As shown in Supplementary Table 4, FSH levels were marginally associated with hippocampal volume across all participants (multivariable adjusted *P* = 0.058). These associations did not reach significance in the postmenopausal or perimenopausal groups. There were no significant associations between LH and hippocampal volume, overall or by menopause status.

#### APOE-4 status effects

APOE-4 status was not significantly associated with regional biomarker outcomes, and did not show interaction effects with hormone-biomarker associations (Table 7). Descriptively, the interaction between LH and APOE-4 status for the frontal GMV outcome was marginally significant (*P* = 0.104). This effect was driven by APOE-4 carriers exhibiting LH-GMV associations [adjusted estimate = −0.08, 95% CI−0.138,−0.021, *P* < 0.01] whereas no effects were observed among noncarriers [adjusted estimate = −0.03, 95% CI−0.081, 0.032, *P* = 0.397].

#### Effects of estradiol

**FIGURE 5:**
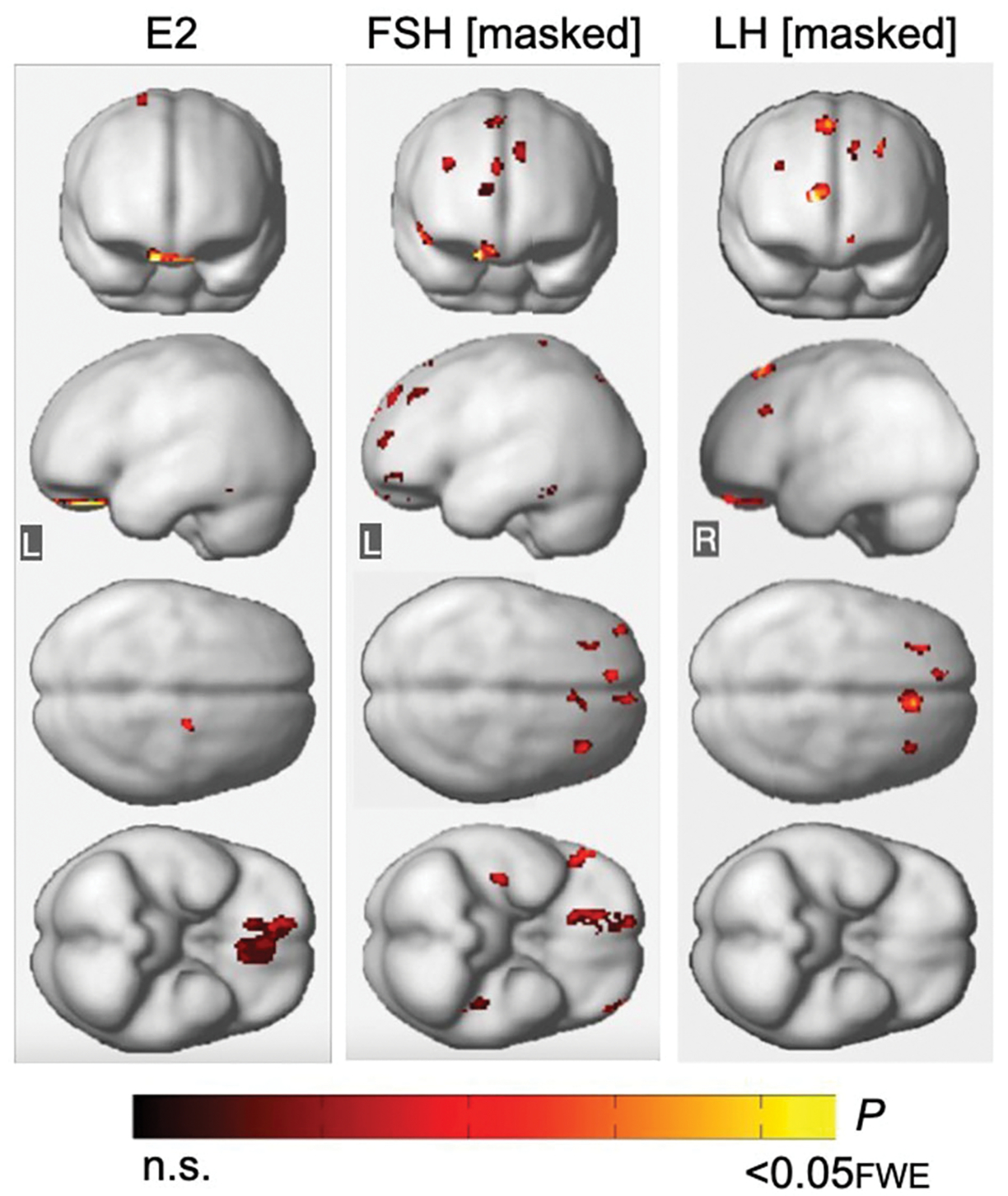
Associations of FSH and LH with gray matter volume excluding regional effects of estradiol levels. **(Left)** Statistical parametric maps (SPMs) displaying positive voxel-wise associations between estradiol (E2) and gray matter volume (GMV), excluding all regional effects of age and menopause. **(Middle)** SPMs displaying negative voxel-wise associations between FSH and GMV, excluding all regions that have significant associations with E2, age and menopause status. **(Right)** SPMs displaying negative voxel-wise associations between LH and GMV, excluding all regions that have significant associations with E2, age and menopause status. SPMs are generated within the prespecified search volume of AD-vulnerable regions including medial temporal lobe and displayed on the anterior, lateral, superior and inferior views of a volume-rendered MR template image. Results are represented on a color-coded scale with corresponding *P*-values. FWE, family wise type errors; L, left; R, right.

Across all participants, adjusting for age and menopause status, there were no associations between estradiol levels and Aβ load in any region. For GMV measures, estradiol levels were positively associated with GMV in orbitofrontal gyrus, right medial frontal gyrus, and left fusiform gyrus (*P*_*FWE*_≤ 0.018; Supplementary Table 5; Figure 5). After excluding MHT users, estradiol levels were associated with GMV in orbitofrontal gyrus, as well as in additional smaller clusters located in bilateral middle frontal gyrus and left parahippocampal gyrus (*P*_*FWE*_≤ 0.015; Supplementary Table 5).

Associations of FSH and LH with GMV remained substantially unchanged after excluding all regions with estradiol-GMV associations in the entire sample (*P*_*FWE*_≤ 0.013 and *P*_*FWE*_≤ 0.003, respectively; Supplementary Table 6; Figure 5), as well as among MHT non-users (*P*_*FWE*_≤ 0.021 and *P*_*FWE*_ = 0.007, respectively, Supplementary Table 6). An area of partial overlap between FSH- and estradiol-related clusters was noted in right orbitofrontal cortex across all women, and in middle frontal gyrus and fusiform gyrus of the left hemisphere among MHT non-users, which resulted in diminished yet significant FSH-GMV associations in those areas (Supplementary Table 6).

### Associations of FSH, LH, and cognition

Adjusting by age and education, there were no significant associations between FSH, LH and global cognition or verbal memory composite scores (Supplementary Table 7).

## Discussion

In this translational neuroimaging study, increasing serum gonadotropin levels across the menopause transition, especially FSH levels, were associated with higher Aβ load and lower GMV in frontal areas of midlife women at risk for AD. Results were independent of age, menopause type, hormone therapy status, history of depression, and APOE-4 status. Gonadotropin effects on biomarkers were evident at the perimenopausal stage and more pronounced at the postmenopausal stage. Associations with Aβ load involved middle and superior frontal gyrus for FSH and inferior frontal gyrus for LH. This aligns with the notion that the Aβ deposition process intensifies in specific areas, such as frontal cortex (Braak et al., 2006; LaFerla et al., 2007; Shankar et al., 2008). Moreover, associations with Aβ load were more robust with FSH than with LH, as the latter did not survive multivariable adjustment and did not reach significance among MHT non-users. This supports evidence of differences in onset and control mechanisms that may account for the initial impact of FSH (Xiong et al., 2022). FSH levels rise before those of LH (Padmanabhan and Cardoso, 2020), partly due to FSH’s greater sensitivity to the inhibition of HPG axis hormones, such as inhibin B, which decreases during early perimenopause independent of changes in estradiol (Padmanabhan and Cardoso, 2020). Further investigation is needed to explore differential trajectories of gonadotropin impacts on AD risk.

Additionally, both FSH and LH showed associations with GMV in frontal regions, consistent with the findings related to Aβ load. FSH also showed borderline associations with hippocampal volume, which were not present for LH. These data may reflect downstream effects of Aβ on neuronal integrity (Klupp et al., 2015), possibly induced by soluble Aβ oligomers promoting neurotoxicity prior to fibrilization into plaques (Fang et al., 2019), but are undetectable by means of ^11^C-PiB PET. Alternatively, gonadotropin-related GMV declines may be due to neurofibrillary tangles, which have been linked to neuronal loss in limbic and temporo-parietal regions prior to Aβ deposition (van der Kant et al., 2020). In a PET study, older postmenopausal women exhibited higher tau deposition in frontal and parieto-occipital regions as compared to age-controlled men, with no differences in cortical Aβ burden (Buckley et al., 2022). No sex differences were observed at the premenopausal stage (Buckley et al., 2022). However, the relationship between tau pathology and hormone levels remains unknown.

Present findings highlight the frontal cortex as a key site for gonadotropic action in brain, consistent with i*n vitro* and *ex vivo* evidence of FSHR and LHR distribution (Ryu et al., 2022; Xiong et al., 2022). Post-mortem studies revealed higher FSHR expression in frontal regions of AD patients compared to controls (Ryu et al., 2022), further implicating FSH in neurological harm. *In vivo* neuroimaging studies also identifies the frontal cortex as an early vulnerability site for both Aβ deposition and GMV loss during the prodromal phase of AD (Teipel et al., 2017; Mosconi et al., 2021). However, it’s important to note that associations between FSH and hippocampal volume were only marginally significant, and LH was not associated with hippocampal volume. Since the hippocampus is considered a region more specifically targeted by AD pathology (Jack et al., 2013), further research is needed to elucidate the precise nature of these associations. Nonetheless, given that the frontal cortex is implicated in both aging and AD, and hormonal fluctuations during menopause impact cellular aging (Brinton et al., 2015), the observed associations may reflect accelerated aging, preclinical AD, or both.

Overall, these findings align with previous research indicating that menopause is a tipping point for AD risk (Brinton et al., 2015), suggesting a window of opportunity for therapeutic intervention (Brinton et al., 2015; Mosconi et al., 2018b). This supports testing of therapies regulating serum gonadotropin levels for AD risk reduction. Estrogen therapy with or without a progestogen is the treatment of choice for menopausal symptoms (Santoro et al., 2021) and holds promise for AD prevention (Levin-Allerhand et al., 2002; Nerattini et al., 2023). Observational studies generally report favorable effects of MHT on AD risk, whereas clinical trials indicate null effects on cognition in early post-menopausal patients and null or negative effects in older post-menopausal women (Maki, 2008; Jett et al., 2022a,b). Protective effects are generally more consistent among younger women, especially following oophorectomy, with benefits pertaining to early initiation relative to surgery or age of menopause onset (Maki, 2013; van der Kant et al., 2020). Notably, in addition to increasing estrogen levels, MHT induces a dose-related decrease in serum gonadotropins (Yen et al., 1975). The neurological effects of these decreases are not well understood. In the Kronos Early Estrogen Prevention Study, FSH decreases following transdermal 17β-estradiol treatment were associated with smaller increases in WMH relative to placebo and oral CEE therapy (Kling et al., 2020). In this study, adjustment for MHT and exclusion of MHT users had little to no impact on GMV results and FSH-Aβ associations, but rendered LH-Aβ associations non-significant. Given the potential impact of FSH on cardiovascular risk (Hyman et al., 2000), more work is warranted to examine whether lowering FSH by means of hormone therapy is a viable strategy to reduce neuronal loss and vascular damage, which are risk factors for AD in turn (Alber et al., 2019).

Estrogen- and progesterone-containing OCP inhibit the release of gonadotropin-releasing hormone (GnRH), suppressing levels of FSH and LH, therefore preventing follicular development and ovulation (D’Arpe et al., 2016). OCP can be used to alleviate symptoms of menopause (Grandi et al., 2022), representing another therapeutic option for controlling gonadotropin effects on AD risk. Generally, MRI studies show greater GMV in OCP users compared to never-users, although results are not always consistent (Jett et al., 2022a). In an MRI study of middle-aged women at risk for AD, those who took OCP before menopause and MHT for menopause exhibited larger GMV in AD-vulnerable regions, including frontal and parietal cortex, medial temporal lobe, precuneus, and fusiform gyrus, as compared to never-users of both (Schelbaum et al., 2021). Notably, these are some of the same regions displaying negative effects of serum gonadotropins on GMV in the present study, warranting further investigation.

Further epidemiological support for a role of gonadotropins in AD is evidenced by a reduction in neurodegenerative disease among prostate cancer patients treated with GnRH agonists (Bowen et al., 2000). GnRH-agonists and antagonists disrupt physiological GnRH pulses, producing a drop in serum FSH and LH levels (Wilson et al., 2007). Preliminary evidence indicates that GnHR agonists reduce Aβ pathology and improved cognition in rodents (Casadesus et al., 2006a). Clinical trials of humanized monoclonal FSH antibodies, which proved effective in reversing AD pathology in mice (Xiong et al., 2022), are also warranted. Additionally, emerging research is investigating whether healthy ovarian tissue cryopreservation may restore pre-menopausal steroid hormone levels in post-menopausal women (Chen et al., 2022; Yoo et al., 2022). Finally, the administration of platelet-rich plasma with recombinant FSH to the ovaries was shown to restore ovarian function, at least temporarily, in early post-menopausal women (Hsu et al., 2021). These techniques aim to extend women’s fertility period by delaying menopause onset. It would be intriguing to explore their potential impact on menopause-associated AD biomarker findings.

### Strengths and limitations

We focused on carefully screened midlife women, ages 40–65 years, with no medical comorbidities or incidental findings. All participants underwent clinical and cognitive exams, laboratory tests, and menopause assessments. From a methodological perspective, we leveraged the menopause transition as a natural experiment of increasing FSH and LH by examining statistically powered groups of women at different menopausal stages. FSH and LH levels are progressively higher from premenopausal through postmenopausal stages, but show uncorrelated patterns and greater differentiation starting at perimenopause. This enabled us to test for differential associations with biomarker outcomes. We used state-of-the-art voxel-based analysis paired with age and menopause correction procedures, stringent statistical reporting criteria, and a translational approach to ensure both statistical and biological validity of our results. Significance was maintained after multivariable correction for age, modality-specific confounders, menopause type, and MHT use.

Our choice of a cross-sectional design was motivated by the high variability in the timing of menopause, with a median age at menopause of 51 years and a range of 40–58 years (Monteleone et al., 2018). Longitudinal studies would require 10 or more years of follow-ups to capture concurrent changes in hormones and brain. Although oophorectomy studies ideally reduce follow-up times, the procedure is often performed due to medical conditions and is associated with more severe neuropathological outcomes than spontaneous menopause (Rocca et al., 2014; Zeydan et al., 2019). However, given the cross-sectional and observational nature of this study, a causal link between gonadotropin levels and brain biomarkers cannot be unequivocally established. Nonetheless, our findings, combined with existing preclinical data demonstrating causative associations between gonadotropin increases, AD pathology and synaptic degeneration (Verdile et al., 2015; Xiong et al., 2022), provide a biological framework for future controlled experimental designs involving manipulation of hormone levels.

Comparisons of FSH, LH and estradiol effects on AD risk have been limited, which may have led to underestimating the role of the gonadotropins. In our study, estradiol levels collected concurrently with FSH and LH were not associated with Aβ load but shown an association with GMV mostly in frontal regions, consistent with the known trophic effects of this hormone on frontal synaptic density (Jett et al., 2022a). Nonetheless, associations between gonadotropin levels and biomarkers were statistically and anatomically independent of estradiol levels, indicating sub-regionally independent effects. Further research is needed to track and compare longitudinal changes in pituitary and ovarian hormones in relation to AD biomarkers and to clarify the clinical implications of these associations.

We did not find significant interactions of APOE-4 status with hormone-biomarker relationships, except for a marginal effect on LH-frontal GMV associations driven by APOE-4 carriers. This suggests that APOE-4′s effects may become pronounced at older ages or with overt disease, or that gonadotropins’ effects on the examined biomarkers occur independently of APOE-4 status. Given the impact of APOE-4 on AD risk (Belloy et al., 2019), future research is warranted to investigate modulatory effects of APOE-4 on gonadotropin-biomarker associations.

Gonadotropin levels did not correlate with global cognition or memory scores. This may be because the postmenopausal group did not exhibit impaired or diminished cognitive performance compared to the other groups, consistent with the observation that, while self-reports of poor memory and concentration are common in women of menopausal age (Monteleone et al., 2018), menopause itself is not associated with clinically significant deficits (Maki and Henderson, 2016). Additionally, our participants had at least 12 years of education, which might have hindered detection of significant associations. It is also possible that our testing battery was not sufficiently demanding to detect early effects of gonadotropin elevations on cognitive function. Additionally, all participants were cognitively normal at the time of evaluation. Conceivably, the associations between gonadotropins and AD biomarkers, especially Aβ deposition, may have effects that take time to manifest. Longitudinal confirmation with multiple assessments in our cohort as well as other studies, is needed to further elucidate the time-dependent associations between gonadotropins and AD-related changes.

Future studies with larger cohorts of midlife women with different educational levels, racial and socioeconomical backgrounds are needed to assess the generalizability of these findings. Clinical application is not yet justified.

## Conclusions

Present results provide evidence for gonadotropin-related AD endophenotypes in midlife women at risk for AD, with serum FSH levels being the main predictor of Aβ load. Outcomes are consistent with preclinical work implicating rising gonadotropin levels in women’s AD risk, and indicate novel hormonal targets for precision medicine strategies for AD risk reduction.

## Supplementary Material

Supplementary Material

## Figures and Tables

**TABLE 1:** Participants’ characteristics.

	Premenopause	Perimenopause	Postmenopause
*N*	45	67	79
Age, years	44 (4)	49 (4)	55 (4)*
Education, years	17 (1)	17 (2)	17 (2)
Race, % white	75	76	82
APOE-4 carrier status, % positive	44	39	50
MoCA score, unitless	28 (1)	29 (2)	29 (1)
History of mild depression, % positive	9	16	13
Menopause hormone therapy, % current users	n.a.	15	34
Oral contraceptives, % current users	4	12	n.a.
Hysterectomy/oophorectomy status, % positive	n.a.	1	22^
**Hormone levels**			
FSH (mIU/mL)	9 (10)	31 (35)*	78 (31)*^
LH (mIU/mL)	8 (6)	20 (19)*	40 (14)*^
Estradiol (pg/mL)	111 (103)	83 (91)	21 (32)*^
**Composite cognitive scores, unitless** ^a^			
Global cognition	0.09 (0.11)	0.08 (0.09)	0.08 (0.08)
Verbal memory	0.13 (0.10)	0.10 (0.08)	0.02 (0.07)

Values are means (standard deviation) unless otherwise indicated.

a Mean (standard error) adjustedby age and years of education.

* Different from pre-menopausal group, p < 0.05

^ Different from peri-menopausal group, p < 0.05.

**TABLE 2:** Associations between FSH and LH levels and Aβ load in AD-regions.

Cluster extent	Coordinates x, y, z	Z	P_FWE_ cluster*	P voxel	Anatomical region	Brodmann area
**FSH associations with Aβ measures**						
49	−47, 28, 33	3.98	0.020	<0.001	Left cerebrum, frontal lobe, middle frontal gyrus	9
	−39, 35, 31	3.42		<0.001	Left cerebrum, frontal lobe, superior frontal gyrus	9
	−41, 27, 42	3.38		<0.001	Left cerebrum, frontal lobe, middle frontal gyrus	8
16	43, 37, 32	3.45	0.028	<0.001	Right cerebrum, frontal lobe, superior frontal gyrus	9
**LH associations with Aβ measures**						
22	57, 37,−8	3.51	0.007	<0.001	Right cerebrum, frontal lobe, inferior frontal gyrus	47

* P<0.05 cluster-level corrected for Family-Type Wise Error (FWE) within the search volume, excluding regional effects of age and menopause status. This was accomplished by using the subtraction analysis approach described in the methods section, where all voxel-wise regional contributions of age and menopause status were excluded from analysis using the explicit masking approach implemented in SPM12.

**TABLE 3:** Multi-variable adjusted associations of FSH and LH with regional Aβ load and gray matter volume.

		Model	Overall			Post			Peri		
			Coeff.	SE	*P*	Coeff.	SE	*P*	Coeff.	SE	*P*
**Aβ load**											
FSH	Middle frontal	1	0.193	0.114	**0.038**	0.305	0.135	**0.038**	0.108	0.182	0.272
		2	0.187	0.116	**0.044**	0.315	0.172	**0.042**	0.112	0.243	0.282
	Superior frontal	1	0.174	0.127	***0.056***	0.021	0.206	0.451	0.259	0.196	***0.069***
		2	0.167	0.129	***0.065***	0.041	0.232	0.414	0.203	0.208	0.145
LH	Inferior frontal	1	0.161	0.111	***0.072***	−0.014	0.249	0.468	0.170	0.170	0.168
		2	0.130	0.109	0.121	−0.076	0.240	0.343	0.166	0.177	0.195
**Gray matter volume**											
FSH	Middle frontal	1	−0.174	0.081	**0.010**	−0.083	0.145	0.242	−0.191	0.127	***0.067***
		2	−0.179	0.082	**0.008**	−0.086	0.147	0.239	−0.202	0.149	*0.070*
	Orbitofrontal	1	−0.246	0.081	**<0.001**	−0.253	0.153	**0.015**	−0.237	0.127	**0.031**
		2	−0.225	0.078	**0.001**	−0.211	0.152	**0.040**	−0.263	0.161	**0.026**
	Inferior frontal	1	−0.265	0.071	**<0.001**	−0.332	0.122	**0.002**	−0.172	0.126	*0.089*
		2	−0.256	0.073	**<0.001**	−0.310	0.130	**0.004**	−0.157	0.143	0.126
	Superior frontal	1	−0.129	0.077	**0.041**	−0.076	0.137	0.259	−0.218	0.128	***0.053***
		2	−0.117	0.077	*0.059*	−0.047	0.133	0.351	−0.191	0.159	*0.082*
LH	Medial frontal	1	−0.276	0.069	**<0.001**	−0.258	0.104	**0.012**	−0.321	0.124	**0.005**
		2	−0.308	0.068	**<0.001**	−0.285	0.097	**0.008**	−0.343	0.143	**0.005**
	Superior frontal	1	−0.250	0.074	**0.001**	−0.402	0.101	**<0.001**	−0.070	0.116	0.294
		2	−0.225	0.071	**0.001**	−0.389	0.097	**<0.001**	−0.031	0.132	0.412

Values are coefficients, standard errors (SE). Biomarker volumes of interest are extracted from peak clusters listed in Tables 2, 5. MRI measures are adjusted by TIV. PiB measures are standardized uptake value ratios (SUVR) to cerebellar gray matter uptake. Significant P-values are in bold; borderline significant P-values are in italics. Model 1: adjustment by age and menopause status; Model 2: further multivariable adjustment. All analyses are adjusted by modality-specific confounders.

**TABLE 4:** Associations between FSH and LH levels with Aβ load in AD-regions among MHT non-users.

Cluster extent	Coordinates x, y, z	Z	P_FWE_ cluster*	P voxel	Anatomical region	Brodmann area
**FSH associations with Aβ measures**						
28	−47, 28, 35	3.99	0.022	<0.001	Left cerebrum, frontal lobe, middle frontal Gyrus	9
	−39, 37, 29	3.29		<0.001	Left cerebrum, frontal lobe, superior frontal gyrus	9
26	43, 37, 32	3.65	0.023	<0.001	Right cerebrum, frontal lobe, superior frontal gyrus	9
**LH associations with Aβ measures**						
n.s.						

* P < 0.05 cluster-level corrected for Family-Type Wise Error (FWE) within the search volume, excluding regional effects of age and menopause status. See legend to Table 2.

**TABLE 5:** Associations between FSH and LH levels with MRI gray matter volume in AD-regions.

Cluster extent	Coordinates x, y, z	Z	P_FWE_ cluster*	P voxel	Anatomical region	Brodmann area
**FSH associations with GMV**						
159	14, 30,−20	4.32	<0.001	<0.001	Right cerebrum, frontal lobe, inferior frontal gyrus	11
	12, 47,−21	4.09		<0.001	Right cerebrum, frontal lobe, orbital gyrus	11
	12, 55,−19	3.59		<0.001	Right cerebrum, frontal lobe, superior frontal gyrus	11
17	−10, 52, 27	3.78	0.016	<0.001	Left cerebrum, frontal lobe, superior frontal gyrus	9
23	35, 33, 26	3.61	0.011	<0.001	Right cerebrum, frontal lobe, middle frontal gyrus	9
52	47, 25,−12	3.47	0.003	<0.001	Right cerebrum, frontal lobe, inferior frontal gyrus	47
	55, 33,−10	3.42		<0.001	Right cerebrum, frontal lobe, inferior frontal gyrus	47
**LH associations with GMV**						
36	10, 35, 46	3.37	0.002	<0.001	Right cerebrum, frontal lobe, superior frontal gyrus	8
22	12, 45, 13	3.69	0.003	<0.001	Right cerebrum, frontal lobe, medial frontal gyrus	10

* P < 0.05 cluster-level corrected for Family-Type Wise Error (FWE) within the search volume, excluding regional effects of age and menopause status. See legend to Table 2.

**TABLE 6:** Associations between FSH and LH levels with MRI gray matter volume in AD-regions among MHT non-users.

Cluster extent	Coordinates x, y, z	Z	P_FWE_ cluster*	P voxel	Anatomical region	Brodmann area
**FSH associations with GMV**						
157	12, 37,−22	4.35	<0.001	<0.001	Right cerebrum, frontal lobe, inferior frontal gyrus	11
	12, 47,−21	4.33		<0.001	Right cerebrum, frontal lobe, orbital gyrus	11
	14, 30,−21	4.28		<0.001	Right cerebrum, frontal lobe, inferior frontal gyrus	11
38	35, 33, 24	4.00	0.009	<0.001	Right cerebrum, frontal lobe, middle frontal gyrus	9
63	−8,−69, 51	3.73	0.004	<0.001	Left cerebrum, parietal lobe, precuneus	7
22	−41,−46,−7	3.56	0.018	<0.001	Left cerebrum, temporal lobe, fusiform gyrus	37
23	−27, 42, 26	3.50	0.017	<0.001	Left cerebrum, frontal lobe, middle frontal gyrus	10
	−29, 34, 36	3.14		<0.001	Left cerebrum, frontal lobe, middle frontal gyrus	9
19	55, 33,−10	3.30	0.021	<0.001	Right cerebrum, frontal lobe, inferior frontal gyrus	47
**LH associations with GMV**						
16	35, 33, 24	3.70	0.007	<0.001	Right cerebrum, frontal lobe, middle frontal gyrus	9
43	12, 47,−21	3.55	0.002	<0.001	Right cerebrum, frontal lobe, orbital gyrus	11
	14, 39,−22	3.46		<0.001	Right cerebrum, frontal lobe, orbital gyrus	11

* P < 0.05 cluster-level corrected for Family-Type Wise Error (FWE) within the search volume, excluding regional effects of age and menopause status. See legend to Table 2.

**TABLE 7:** Effects of APOE-4 status on hormone-biomarker associations.

Exposure	Outcome*	APOE-4 status			Interaction		
		Coeff.	SE	*P*	Coeff.	SE	*P*
FSH	Frontal Aβ load	0.100	0.175	0.986	−0.329	0.193	0.323
	Frontal GMV	0.003	0.025	0.909	0.002	0.026	0.943
LH	Frontal Aβ load	0.130	0.169	0.443	−0.211	0.197	0.287
	Frontal GMV	−0.034	0.034	0.321	−0.088	0.035	0.104

Values are coefficients, standard errors (SE).

* Biomarker data extracted from the peak clusters of statistical significance listed in Tables 2, 5. MRI measures are adjusted byTIV. PiB measures are standardized uptake value ratios (SUVR) to cerebellar gray matter uptake.

## Data Availability

The original contributions presented in the study are included in the article/Supplementary material, further inquiries can be directed to the corresponding author.
