# Supplementary material for: Elevated gonadotropin levels are associated with increased biomarker risk of Alzheimer’s disease in midlife women
Source: Front Dement. Author manuscript; Available in PMC 2024 May 21. (PMC11108587; doi:10.3389/frdem.2023.1303256)
Supplement: Supplementary Material [file NIHMS1980366-supplement-Supplementary_Material.docx]

## e-Table 1. Voxel-based associations of age and menopause with Aβ load in AD-regions

| Cluster extent | Coordinates  x, y, z | Z | P_FWE_ cluster* | P voxel | Anatomical Region | Brodmann Area |
| --- | --- | --- | --- | --- | --- | --- |
| **Association of age and Aβ load** | | | | | | |
| 1083 | 24 -70 51 | 5.14 | <0.001 | <0.001 | Right Cerebrum, Parietal Lobe, Precuneus | 7 |
|  | 51 -58 43 | 5.01 |  | <0.001 | Right Cerebrum, Parietal Lobe, Inferior Parietal Lobule | 40 |
|  | 57 -57 36 | 4.68 |  | <0.001 | Right Cerebrum, Parietal Lobe, Angular Gyrus | 40 |
| 581 | -55 38 -2 | 4.41 | 0.001 | <0.001 | Left Cerebrum, Frontal Lobe, Inferior Frontal Gyrus | 10 |
|  | -45 39 25 | 4.32 |  | <0.001 | Left Cerebrum, Frontal Lobe, Middle Frontal Gyrus | 46 |
|  | -47 50 -8 | 4.10 |  | <0.001 | Left Cerebrum, Frontal Lobe, Middle Frontal Gyrus | 10 |
| 260 | -12 -54 66 | 4.06 | 0.011 | <0.001 | Left Cerebrum, Parietal Lobe, Postcentral Gyrus | 7 |
|  | -22 -60 59 | 4.01 |  | <0.001 | Left Cerebrum, Parietal Lobe, Superior Parietal Lobule | 7 |
| 314 | 57 36 -3 | 3.99 | 0.007 | <0.001 | Right Cerebrum, Frontal Lobe, Inferior Frontal Gyrus | 47 |
|  | 59 26 -7 | 3.72 |  | <0.001 | Right Cerebrum, Frontal Lobe, Inferior Frontal Gyrus | 47 |
|  | 45 55 1 | 3.64 |  | <0.001 | Right Cerebrum, Frontal Lobe, Inferior Frontal Gyrus | 10 |
| **Associations of menopause status and Aβ load** | | | | | | |
| 44 | -29 30 48 | 3.67 | 0.006 | <0.001 | Left Cerebrum, Frontal Lobe, Superior Frontal Gyrus | 8 |

**P* < 0.05 cluster-level corrected for Family-Type Wise Error (FWE) within the search volume. Results are normalized by cerebellar gray matter PiB uptake.

## e-Table 2. Voxel-based associations of age and menopause with MRI gray matter volume in AD-regions

| Cluster extent | Coordinates x, y, z | | Z | | P_FWE_ cluster* | | | P voxel | | Anatomical Region | Brodmann Area |
| --- | --- | --- | --- | --- | --- | --- | --- | --- | --- | --- | --- |
| **Associations of age and gray matter volume** | | | | | | | | | | | |
| 146 | 47 18 44 | | 3.66 | | <0.001 | | | <0.001 | | Right Cerebrum, Frontal Lobe, Middle Frontal Gyrus | 8 |
| 51 | -47 6 20 | | 4.43 | | 0.040 | | | <0.001 | | Left Cerebrum, Frontal Lobe, Inferior Frontal Gyrus | 44 |
|  | -53 7 15 | | 4.10 | |  | | | <0.001 | | Left Cerebrum, Frontal Lobe, Inferior Frontal Gyrus | 44 |
| 274 | -8 -29 30 | | 4.36 | | <0.001 | | | <0.001 | | Left Cerebrum, Limbic Lobe, Cingulate Gyrus | 23 |
|  | 10 -25 30 | | 4.04 | |  | | | <0.001 | | Right Cerebrum, Limbic Lobe, Cingulate Gyrus | 23 |
|  | -6 -16 29 | | 3.94 | |  | | | <0.001 | | Left Cerebrum, Limbic Lobe, Cingulate Gyrus | 23 |
| 774 | 59 2 -2 | | 4.30 | | <0.001 | | | <0.001 | | Right Cerebrum, Temporal Lobe, Superior Temporal Gyrus | 22 |
|  | 57 25 23 | | 4.28 | |  | | | <0.001 | | Right Cerebrum, Frontal Lobe, Middle Frontal Gyrus | 46 |
|  | 57 -25 13 | | 4.18 | |  | | | <0.001 | | Right Cerebrum, Temporal Lobe, Superior Temporal Gyrus | 41 |
| 51 | 8 -6 16 | | 4.20 | | 0.040 | | | <0.001 | | Right Cerebrum, Sub-lobar, Thalamus |  |
|  | 4 -12 13 | | 3.30 | |  | | | <0.001 | | Right Cerebrum, Sub-lobar, Thalamus |  |
| 62 | -12 -37 7 | | 4.11 | | 0.028 | | | <0.001 | | Left Cerebrum, Limbic Lobe, Parahippocampal Gyrus | 30 |
|  | -18 -33 12 | | 3.25 | |  | | | <0.001 | | Left Cerebrum, Sub-lobar, Thalamus |  |
| 157 | -59 -4 0 | | 3.92 | | 0.002 | | | <0.001 | | Left Cerebrum, Temporal Lobe, Superior Temporal Gyrus | 22 |
|  | -55 -15 1 | | 3.52 | |  | | | <0.001 | | Left Cerebrum, Temporal Lobe, Superior Temporal Gyrus | 22 |
|  | -61 -16 10 | | 3.51 | |  | | | <0.001 | | Left Cerebrum, Temporal Lobe, Transverse Temporal Gyrus | 42 |
| 56 | -49 29 11 | | 3.85 | | 0.034 | | | <0.001 | | Left Cerebrum, Frontal Lobe, Inferior Frontal Gyrus | 46 |
|  | -6 6 23 | | 3.16 | |  | | | <0.001 | | Left Cerebrum, Limbic Lobe, Cingulate Gyrus | 24 |
| 50 | -57 -31 10 | | 3.71 | | 0.042 | | | <0.001 | | Left Cerebrum, Temporal Lobe, Superior Temporal Gyrus | 42 |
| 71 | 69 -25 -2 | | 3.40 | | 0.021 | | | <0.001 | | Right Cerebrum, Temporal Lobe, Middle Temporal Gyrus | 21 |
|  | 63 -31 -6 | | 3.33 | |  | | | <0.001 | | Right Cerebrum, Temporal Lobe, Middle Temporal Gyrus | 21 |
| **Associations of menopause status and gray matter volume** | | | | | | | | | | | |
| 18 | | 10 -32 39 | | 3.73 | | 0.003 | <0.001 | | Right Cerebrum, Limbic Lobe, Cingulate Gyrus | | 31 |
| 23 | | 20 -49 5 | | 3.73 | | 0.003 | <0.001 | | Right Cerebrum, Limbic Lobe, Parahippocampal Gyrus | | 30 |
| 19 | | 10 -45 23 | | 3.57 | | 0.004 | <0.001 | | Right Cerebrum, Limbic Lobe, Posterior Cingulate | | 30 |

**P* < 0.05 cluster-level corrected for Family-Type Wise Error (FWE) within the search volume. Analyses are adjusted by total intracranial volume. Associations of menopause status and gray matter volume are further adjusted by age.

## e-Table 3. Voxel-based associations of FSH and LH with MRI gray matter volume at the whole brain level.

| Cluster extent | Coordinates  x, y, z | Z | P_FWE_ cluster* | P voxel | Anatomical Region | Brodmann Area |
| --- | --- | --- | --- | --- | --- | --- |
| **FSH associations with GMV among all women** | | | | | | |
| 202 | 14 30 -20 | 4.32 | <0.001 | <0.001 | Right Cerebrum, Frontal Lobe, Inferior Frontal Gyrus | 11 |
|  | 12 47 -21 | 4.09 |  | <0.001 | Right Cerebrum, Frontal Lobe, Orbital Gyrus | 11 |
|  | 12 55 -19 | 3.59 |  | <0.001 | Right Cerebrum, Frontal Lobe, Superior Frontal Gyrus | 11 |
| 17 | -10 52 27 | 3.78 | 0.025 | <0.001 | Left Cerebrum, Frontal Lobe, Superior Frontal Gyrus | 9 |
| 58 | 4 15 53 | 3.71 | 0.006 | <0.001 | Right Cerebrum, Frontal Lobe, Superior Frontal Gyrus | 6 |
|  | 4 26 49 | 3.41 |  | <0.001 | Right Cerebrum, Frontal Lobe, Superior Frontal Gyrus | 8 |
| 24 | 35 28 1 | 3.71 | 0.019 | <0.001 | Right Cerebrum, Frontal Lobe, Inferior Frontal Gyrus | 47 |
| 23 | 35 33 26 | 3.61 | 0.019 | <0.001 | Right Cerebrum, Frontal Lobe, Middle Frontal Gyrus | 9 |
| 38 | 47 25 -12 | 3.47 | 0.011 | <0.001 | Right Cerebrum, Frontal Lobe, Inferior Frontal Gyrus | 47 |
|  | 53 33 -10 | 3.34 |  | <0.001 | Right Cerebrum, Frontal Lobe, Inferior Frontal Gyrus | 47 |
| 16 | -31 21 6 | 3.41 | 0.026 | <0.001 | Left Cerebrum, Sub-lobar, Insula | 13 |
| **FSH associations with GMV among MHT non-users** | | | | | | |
| 263 | 12 37 -22 | 4.35 | <0.001 | <0.001 | Right Cerebrum, Frontal Lobe, Inferior Frontal Gyrus | 11 |
|  | 12 47 -21 | 4.33 |  | <0.001 | Right Cerebrum, Frontal Lobe, Orbital Gyrus | 11 |
|  | 14 30 -21 | 4.28 |  | <0.001 | Right Cerebrum, Frontal Lobe, Inferior Frontal Gyrus | 11 |
| 38 | 35 33 24 | 4.00 | 0.016 | <0.001 | Right Cerebrum, Frontal Lobe, Middle Frontal Gyrus | 9 |
| 81 | 4 17 53 | 3.80 | 0.004 | <0.001 | Right Cerebrum, Frontal Lobe, Superior Frontal Gyrus | 6 |
|  | 4 26 49 | 3.32 |  | <0.001 | Right Cerebrum, Frontal Lobe, Superior Frontal Gyrus | 8 |
| 59 | -8 -69 51 | 3.73 | 0.008 | <0.001 | Left Cerebrum, Parietal Lobe, Precuneus | 7 |
| 21 | 35 27 1 | 3.70 | 0.030 | <0.001 | Right Cerebrum, Frontal Lobe, Inferior Frontal Gyrus | 47 |
| 22 | -41 -46 -7 | 3.56 | 0.029 | <0.001 | Left Cerebrum, Temporal Lobe, Fusiform Gyrus | 37 |
| 23 | -27 42 26 | 3.50 | 0.028 | <0.001 | Left Cerebrum, Frontal Lobe, Middle Frontal Gyrus | 10 |
|  | -29 34 36 | 3.14 |  | <0.001 | Left Cerebrum, Frontal Lobe, Middle Frontal Gyrus | 9 |
| 16 | 2 18 -23 | 3.24 | 0.037 | <0.001 | Right Cerebrum, Frontal Lobe, Rectal Gyrus | 11 |
| **LH associations with GMV among all women** | | | | | | |
| 25 | 12 45 13 | 3.69 | 0.008 | <0.001 | Right Cerebrum, Frontal Lobe, Medial Frontal Gyrus | 10 |
| 17 | 35 -5 -14 | 3.68 | 0.011 | <0.001 | Right Cerebrum, Limbic Lobe, Parahippocampal Gyrus | Amygdala |
| 49 | 0 -76 -17 | 3.52 | 0.004 | <0.001 | Right Cerebellum, Posterior Lobe, Declive of Vermis | * |
| 50 | 10 35 46 | 3.37 | 0.003 | <0.001 | Right Cerebrum, Frontal Lobe, Superior Frontal Gyrus | 8 |
|  | 2 34 51 | 3.31 |  | <0.001 | Right Cerebrum, Frontal Lobe, Superior Frontal Gyrus | 8 |
| **LH associations with GMV among MHT non-users** | | | | | | |
| 16 | 35 33 24 | 3.70 | 0.010 | <0.001 | Right Cerebrum, Frontal Lobe, Middle Frontal Gyrus | 9 |
| 44 | 12 47 -21 | 3.55 | 0.003 | <0.001 | Right Cerebrum, Frontal Lobe, Orbital Gyrus | 11 |
|  | 14 39 -22 | 3.46 |  | <0.001 | Right Cerebrum, Frontal Lobe, Orbital Gyrus | 11 |
| 28 | 61 -23 -21 | 3.51 | 0.006 | <0.001 | Right Cerebrum, Temporal Lobe, Inferior Temporal Gyrus | 20 |

**P*<0.05 cluster-level corrected for Family-Type Wise Error (FWE) within the search volume, adjusting by total intracranial volume. Analyses were conducted using the subtraction analysis approach described in the methods section, where all voxel-wise regional contributions of age and menopause status were excluded from analysis using the explicit masking approach implemented in SPM12.

## e-Table 4. Associations of FSH and LH levels with hippocampal volume

|  |  | **Overall** | | | **Postmenopause** | | | **Perimenopause** | | |
| --- | --- | --- | --- | --- | --- | --- | --- | --- | --- | --- |
|  | Model | Coeff. | SE | *P* | Coeff. | SE | *P* | Coeff. | SE | *P* |
| FSH | 1 | -0.107 | 0.083 | *0.077* | -0.153 | 0.114 | *0.098* | -0.093 | 0.132 | 0.235 |
|  | 2 | -0.118 | 0.081 | *0.058* | -0.143 | 0.116 | 0.119 | -0.139 | 0.137 | 0.155 |
| LH | 1 | -0.034 | 0.083 | 0.180 | -0.040 | 0.113 | 0.367 | -0.014 | 0.150 | 0.456 |
|  | 2 | -0.047 | 0.082 | 0.266 | -0.036 | 0.119 | 0.382 | -0.044 | 0.157 | 0.375 |

Values are coefficients, standard errors (SE).

Borderline significant *P* values are in italics. Model 1: adjustment by age and menopause status; Model 2: further multivariable adjustment. All analyses are adjusted by total intracranial volume.

## e-Table 5. Associations of estradiol levels with MRI gray matter volume in AD-regions

| Cluster extent | Coordinates x, y, z | Z | P_FWE_ cluster* | P voxel | Anatomical Region | Brodmann Area |
| --- | --- | --- | --- | --- | --- | --- |
| **Overall** | | | | | | |
| 236 | 12 47 -21 | 3.82 | <0.001 | <0.001 | Right Cerebrum, Frontal Lobe, Orbital Gyrus | 11 |
|  | -2 32 -21 | 3.78 |  | <0.001 | Left Cerebrum, Frontal Lobe, Rectal Gyrus | 11 |
|  | 12 30 -21 | 3.66 |  | <0.001 | Right Cerebrum, Frontal Lobe, Rectal Gyrus | 11 |
| 23 | 10 42 -10 | 3.76 | 0.015 | <0.001 | Right Cerebrum, Frontal Lobe, Medial Frontal Gyrus | 10 |
| 20 | -39 -50 -8 | 3.32 | 0.018 | <0.001 | Left Cerebrum, Temporal Lobe, Fusiform Gyrus | 37 |
| **MHT non-users** | | | | | | |
| 45 | 12 47 -21 | 3.80 | 0.006 | 0.000 | Right Cerebrum, Frontal Lobe, Orbital Gyrus | 11 |
| 37 | -39 -44 -7 | 3.74 | 0.008 | 0.000 | Left Cerebrum, Limbic Lobe, Parahippocampal Gyrus | 19 |
| 33 | -27 44 26 | 3.68 | 0.009 | 0.000 | Left Cerebrum, Frontal Lobe, Middle Frontal Gyrus | 10 |
| 77 | 0 37 -22 | 3.66 | 0.002 | 0.000 | Left Cerebrum, Frontal Lobe, Rectal Gyrus | 11 |
|  | -2 21 -20 | 3.58 |  | 0.000 | Left Cerebrum, Frontal Lobe, Rectal Gyrus | 11 |
| 21 | 33 34 36 | 3.61 | 0.015 | 0.000 | Right Cerebrum, Frontal Lobe, Middle Frontal Gyrus | 9 |

**P*<0.05 cluster-level corrected for Family-Type Wise Error (FWE) within the search volume, adjusting by total intracranial volume. Analyses were conducted using the subtraction analysis approach described in the methods section, where all voxel-wise regional contributions of age and menopause status were excluded from analysis using the explicit masking approach implemented in SPM12.

## e-Table 6. Voxel-based associations of FSH and LH with MRI gray matter volume excluding regional effects of estradiol

| Cluster extent | Coordinates x, y, z | Z | P_FWE_ cluster* | P voxel | Anatomical Region | Brodmann Area |
| --- | --- | --- | --- | --- | --- | --- |
| **Overall FSH associations with GMV** | | | | | | |
| 67 | 14 32 -20 | 4.21 | 0.002 | <0.001 | Right Cerebrum, Frontal Lobe, Inferior Frontal Gyrus | 11 |
|  | 10 49 -20 | 3.91 |  | <0.001 | Right Cerebrum, Frontal Lobe, Orbital Gyrus | 11 |
|  | 12 57 -18 | 3.48 |  | <0.001 | Right Cerebrum, Frontal Lobe, Superior Frontal Gyrus | 11 |
| 17 | -10 52 27 | 3.78 | 0.013 | <0.001 | Left Cerebrum, Frontal Lobe, Superior Frontal Gyrus | 9 |
| 23 | 35 33 26 | 3.61 | 0.010 | <0.001 | Right Cerebrum, Frontal Lobe, Middle Frontal Gyrus | 9 |
| 52 | 47 25 -12 | 3.47 | 0.003 | <0.001 | Right Cerebrum, Frontal Lobe, Inferior Frontal Gyrus | 47 |
|  | 55 33 -10 | 3.42 |  | <0.001 | Right Cerebrum, Frontal Lobe, Inferior Frontal Gyrus | 47 |
| **Overall LH associations with GMV** | | | | | | |
| 22 | 12 45 13 | 3.69 | 0.003 | <0.001 | Right Cerebrum, Frontal Lobe, Medial Frontal Gyrus | 10 |
| 36 | 10 35 46 | 3.37 | 0.002 | <0.001 | Right Cerebrum, Frontal Lobe, Superior Frontal Gyrus | 8 |
| **FSH associations with GMV among MHT non-users** | | | | | | |
| 112 | 14 30 -21 | 4.28 | 0.001 | <0.001 | Right Cerebrum, Frontal Lobe, Inferior Frontal Gyrus | 11 |
|  | 10 49 -20 | 3.88 |  | <0.001 | Right Cerebrum, Frontal Lobe, Orbital Gyrus | 11 |
| 38 | 35 33 24 | 4.00 | 0.009 | <0.001 | Right Cerebrum, Frontal Lobe, Middle Frontal Gyrus | 9 |
| 63 | -8 -69 51 | 3.73 | 0.004 | <0.001 | Left Cerebrum, Parietal Lobe, Precuneus | 7 |
| 19 | 55 33 -10 | 3.30 | 0.021 | <0.001 | Right Cerebrum, Frontal Lobe, Inferior Frontal Gyrus | 47 |
| **LH associations with GMV among MHT non-users** | | | | | | |
| 16 | 35 33 24 | 3.70 | 0.007 | <0.001 | Right Cerebrum, Frontal Lobe, Middle Frontal Gyrus | 9 |

**P*<0.05 cluster-level corrected for Family-Type Wise Error (FWE) within the search volume, adjusting by total intracranial volume. Analyses were conducted using the subtraction analysis approach described in the methods section, where all voxel-wise regional contributions of age and menopause status were excluded from analysis using the explicit masking approach implemented in SPM12.

## e-Table 7. Associations between FSH, LH and cognitive scores

|  | **FSH** | | | **LH** | | |
| --- | --- | --- | --- | --- | --- | --- |
|  | Coeff. | Std. Err. | *P* | Coeff. | Std. Err. | *P* |
| **Overall** |  |  |  |  |  |  |
| global cognition | -0.061 | 0.057 | 0.426 | -0.021 | 0.057 | 0.781 |
| memory | -0.057 | 0.052 | 0.448 | -0.037 | 0.052 | 0.617 |
| **Post-menopause** |  |  |  |  |  |  |
| global cognition | -0.163 | 0.125 | 0.199 | 0.057 | 0.122 | 0.630 |
| memory | -0.144 | 0.113 | 0.207 | 0.043 | 0.108 | 0.711 |
| **Peri-menopause** |  |  |  |  |  |  |
| global cognition | -0.063 | 0.115 | 0.628 | -0.083 | 0.101 | 0.524 |
| memory | -0.068 | 0.105 | 0.518 | -0.108 | 0.093 | 0.396 |
| **Pre-menopause** |  |  |  |  |  |  |
| global cognition | -0.033 | 0.193 | 0.840 | -0.143 | 0.202 | 0.380 |
| memory | 0.082 | 0.187 | 0.612 | -0.060 | 0.196 | 0.710 |

Values are coefficients, standard errors (SE). Results are adjusted by age and education.
