# Supplementary material for: Elevated gonadotropin levels are associated with increased biomarker risk of Alzheimer's disease in midlife women
Source: Front Dement. 2023 Nov 23;2:1303256. doi: 10.3389/frdem.2023.1303256 (PMC11108587; doi:10.3389/frdem.2023.1303256)
Supplement: Supplementary file 1 [file Table_1.DOCX]

## e-Table 1. Voxel-based associations of age and menopause with Aβ load in AD-regions 

| Cluster extent | Coordinates x, y, z | Z | P_FWE_ cluster* | P voxel | Anatomical Region | Brodmann Area |
| --- | --- | --- | --- | --- | --- | --- |
| **Association of age and Aβ load** |  |  |  |  |  |  |
| 1083 | 24 -70 51 | 5.14 | <0.001 | <0.001 | Right Cerebrum, Parietal Lobe, Precuneus | 7 |
|  | 51 -58 43 | 5.01 |  | <0.001 | Right Cerebrum, Parietal Lobe, Inferior Parietal Lobule | 40 |
|  | 57 -57 36 | 4.68 |  | <0.001 | Right Cerebrum, Parietal Lobe, Angular Gyrus | 40 |
| 581 | -55 38 -2 | 4.41 | 0.001 | <0.001 | Left Cerebrum, Frontal Lobe, Inferior Frontal Gyrus | 10 |
|  | -45 39 25 | 4.32 |  | <0.001 | Left Cerebrum, Frontal Lobe, Middle Frontal Gyrus | 46 |
|  | -47 50 -8 | 4.10 |  | <0.001 | Left Cerebrum, Frontal Lobe, Middle Frontal Gyrus | 10 |
| 260 | -12 -54 66 | 4.06 | 0.011 | <0.001 | Left Cerebrum, Parietal Lobe, Postcentral Gyrus | 7 |
|  | -22 -60 59 | 4.01 |  | <0.001 | Left Cerebrum, Parietal Lobe, Superior Parietal Lobule | 7 |
| 314 | 57 36 -3 | 3.99 | 0.007 | <0.001 | Right Cerebrum, Frontal Lobe, Inferior Frontal Gyrus | 47 |
|  | 59 26 -7 | 3.72 |  | <0.001 | Right Cerebrum, Frontal Lobe, Inferior Frontal Gyrus | 47 |
|  | 45 55 1 | 3.64 |  | <0.001 | Right Cerebrum, Frontal Lobe, Inferior Frontal Gyrus | 10 |
| **Associations of menopause status and Aβ load** |  |  |  |  |  |  |
| 44 | -29 30 48 | 3.67 | 0.006 | <0.001 | Left Cerebrum, Frontal Lobe, Superior Frontal Gyrus | 8 |

**P* < 0.05 cluster-level corrected for Family-Type Wise Error (FWE) within the search volume. Results are normalized by cerebellar gray matter PiB uptake.
