# Supplementary material for: Elevated gonadotropin levels are associated with increased biomarker risk of Alzheimer's disease in midlife women
Source: Front Dement. 2023 Nov 23;2:1303256. doi: 10.3389/frdem.2023.1303256 (PMC11108587; doi:10.3389/frdem.2023.1303256)
Supplement: Supplementary file 2 [file Table_2.DOCX]

## e-Table 2. Voxel-based associations of age and menopause with MRI gray matter volume in AD-regions

 

| Cluster extent | Coordinates x, y, z | Z | P_FWE_ cluster* | P voxel | Anatomical Region | Brodmann Area |
| --- | --- | --- | --- | --- | --- | --- |
| **Associations of age and gray matter volume** |  |  |  |  |  |  |
| 146 | 47 18 44 | 3.66 | <0.001 | <0.001 | Right Cerebrum, Frontal Lobe, Middle Frontal Gyrus | 8 |
| 51 | -47 6 20 | 4.43 | 0.040 | <0.001 | Left Cerebrum, Frontal Lobe, Inferior Frontal Gyrus | 44 |
|  | -53 7 15 | 4.10 |  | <0.001 | Left Cerebrum, Frontal Lobe, Inferior Frontal Gyrus | 44 |
| 274 | -8 -29 30 | 4.36 | <0.001 | <0.001 | Left Cerebrum, Limbic Lobe, Cingulate Gyrus | 23 |
|  | 10 -25 30 | 4.04 |  | <0.001 | Right Cerebrum, Limbic Lobe, Cingulate Gyrus | 23 |
|  | -6 -16 29 | 3.94 |  | <0.001 | Left Cerebrum, Limbic Lobe, Cingulate Gyrus | 23 |
| 774 | 59 2 -2 | 4.30 | <0.001 | <0.001 | Right Cerebrum, Temporal Lobe, Superior Temporal Gyrus | 22 |
|  | 57 25 23 | 4.28 |  | <0.001 | Right Cerebrum, Frontal Lobe, Middle Frontal Gyrus | 46 |
|  | 57 -25 13 | 4.18 |  | <0.001 | Right Cerebrum, Temporal Lobe, Superior Temporal Gyrus | 41 |
| 51 | 8 -6 16 | 4.20 | 0.040 | <0.001 | Right Cerebrum, Sub-lobar, Thalamus |  |
|  | 4 -12 13 | 3.30 |  | <0.001 | Right Cerebrum, Sub-lobar, Thalamus |  |
| 62 | -12 -37 7 | 4.11 | 0.028 | <0.001 | Left Cerebrum, Limbic Lobe, Parahippocampal Gyrus | 30 |
|  | -18 -33 12 | 3.25 |  | <0.001 | Left Cerebrum, Sub-lobar, Thalamus |  |
| 157 | -59 -4 0 | 3.92 | 0.002 | <0.001 | Left Cerebrum, Temporal Lobe, Superior Temporal Gyrus | 22 |
|  | -55 -15 1 | 3.52 |  | <0.001 | Left Cerebrum, Temporal Lobe, Superior Temporal Gyrus | 22 |
|  | -61 -16 10 | 3.51 |  | <0.001 | Left Cerebrum, Temporal Lobe, Transverse Temporal Gyrus | 42 |
| 56 | -49 29 11 | 3.85 | 0.034 | <0.001 | Left Cerebrum, Frontal Lobe, Inferior Frontal Gyrus | 46 |
|  | -6 6 23 | 3.16 |  | <0.001 | Left Cerebrum, Limbic Lobe, Cingulate Gyrus | 24 |
| 50 | -57 -31 10 | 3.71 | 0.042 | <0.001 | Left Cerebrum, Temporal Lobe, Superior Temporal Gyrus | 42 |
| 71 | 69 -25 -2 | 3.40 | 0.021 | <0.001 | Right Cerebrum, Temporal Lobe, Middle Temporal Gyrus | 21 |
|  | 63 -31 -6 | 3.33 |  | <0.001 | Right Cerebrum, Temporal Lobe, Middle Temporal Gyrus | 21 |
| **Associations of menopause status and gray matter volume** |  |  |  |  |  |  |
| 18 | 10 -32 39 | 3.73 | 0.003 | <0.001 | Right Cerebrum, Limbic Lobe, Cingulate Gyrus | 31 |
| 23 | 20 -49 5 | 3.73 | 0.003 | <0.001 | Right Cerebrum, Limbic Lobe, Parahippocampal Gyrus | 30 |
| 19 | 10 -45 23 | 3.57 | 0.004 | <0.001 | Right Cerebrum, Limbic Lobe, Posterior Cingulate | 30 |

**P* < 0.05 cluster-level corrected for Family-Type Wise Error (FWE) within the search volume. Analyses are adjusted by total intracranial volume. Associations of menopause status and gray matter volume are further adjusted by age.
