# Supplementary material for: Elevated gonadotropin levels are associated with increased biomarker risk of Alzheimer's disease in midlife women
Source: Front Dement. 2023 Nov 23;2:1303256. doi: 10.3389/frdem.2023.1303256 (PMC11108587; doi:10.3389/frdem.2023.1303256)
Supplement: Supplementary file 3 [file Table_3.DOCX]

## e-Table 3. Voxel-based associations of FSH and LH with MRI gray matter volume at the whole brain level.

| Cluster extent | Coordinates x, y, z | Z | P_FWE_ cluster* | P voxel | Anatomical Region | Brodmann Area |
| --- | --- | --- | --- | --- | --- | --- |
| **FSH associations with GMV among all women** |  |  |  |  |  |  |
| 202 | 14 30 -20 | 4.32 | <0.001 | <0.001 | Right Cerebrum, Frontal Lobe, Inferior Frontal Gyrus | 11 |
|  | 12 47 -21 | 4.09 |  | <0.001 | Right Cerebrum, Frontal Lobe, Orbital Gyrus | 11 |
|  | 12 55 -19 | 3.59 |  | <0.001 | Right Cerebrum, Frontal Lobe, Superior Frontal Gyrus | 11 |
| 17 | -10 52 27 | 3.78 | 0.025 | <0.001 | Left Cerebrum, Frontal Lobe, Superior Frontal Gyrus | 9 |
| 58 | 4 15 53 | 3.71 | 0.006 | <0.001 | Right Cerebrum, Frontal Lobe, Superior Frontal Gyrus | 6 |
|  | 4 26 49 | 3.41 |  | <0.001 | Right Cerebrum, Frontal Lobe, Superior Frontal Gyrus | 8 |
| 24 | 35 28 1 | 3.71 | 0.019 | <0.001 | Right Cerebrum, Frontal Lobe, Inferior Frontal Gyrus | 47 |
| 23 | 35 33 26 | 3.61 | 0.019 | <0.001 | Right Cerebrum, Frontal Lobe, Middle Frontal Gyrus | 9 |
| 38 | 47 25 -12 | 3.47 | 0.011 | <0.001 | Right Cerebrum, Frontal Lobe, Inferior Frontal Gyrus | 47 |
|  | 53 33 -10 | 3.34 |  | <0.001 | Right Cerebrum, Frontal Lobe, Inferior Frontal Gyrus | 47 |
| 16 | -31 21 6 | 3.41 | 0.026 | <0.001 | Left Cerebrum, Sub-lobar, Insula | 13 |
| **FSH associations with GMV among MHT non-users** |  |  |  |  |  |  |
| 263 | 12 37 -22 | 4.35 | <0.001 | <0.001 | Right Cerebrum, Frontal Lobe, Inferior Frontal Gyrus | 11 |
|  | 12 47 -21 | 4.33 |  | <0.001 | Right Cerebrum, Frontal Lobe, Orbital Gyrus | 11 |
|  | 14 30 -21 | 4.28 |  | <0.001 | Right Cerebrum, Frontal Lobe, Inferior Frontal Gyrus | 11 |
| 38 | 35 33 24 | 4.00 | 0.016 | <0.001 | Right Cerebrum, Frontal Lobe, Middle Frontal Gyrus | 9 |
| 81 | 4 17 53 | 3.80 | 0.004 | <0.001 | Right Cerebrum, Frontal Lobe, Superior Frontal Gyrus | 6 |
|  | 4 26 49 | 3.32 |  | <0.001 | Right Cerebrum, Frontal Lobe, Superior Frontal Gyrus | 8 |
| 59 | -8 -69 51 | 3.73 | 0.008 | <0.001 | Left Cerebrum, Parietal Lobe, Precuneus | 7 |
| 21 | 35 27 1 | 3.70 | 0.030 | <0.001 | Right Cerebrum, Frontal Lobe, Inferior Frontal Gyrus | 47 |
| 22 | -41 -46 -7 | 3.56 | 0.029 | <0.001 | Left Cerebrum, Temporal Lobe, Fusiform Gyrus | 37 |
| 23 | -27 42 26 | 3.50 | 0.028 | <0.001 | Left Cerebrum, Frontal Lobe, Middle Frontal Gyrus | 10 |
|  | -29 34 36 | 3.14 |  | <0.001 | Left Cerebrum, Frontal Lobe, Middle Frontal Gyrus | 9 |
| 16 | 2 18 -23 | 3.24 | 0.037 | <0.001 | Right Cerebrum, Frontal Lobe, Rectal Gyrus | 11 |
| **LH associations with GMV among all women** |  |  |  |  |  |  |
| 25 | 12 45 13 | 3.69 | 0.008 | <0.001 | Right Cerebrum, Frontal Lobe, Medial Frontal Gyrus | 10 |
| 17 | 35 -5 -14 | 3.68 | 0.011 | <0.001 | Right Cerebrum, Limbic Lobe, Parahippocampal Gyrus | Amygdala |
| 49 | 0 -76 -17 | 3.52 | 0.004 | <0.001 | Right Cerebellum, Posterior Lobe, Declive of Vermis | * |
| 50 | 10 35 46 | 3.37 | 0.003 | <0.001 | Right Cerebrum, Frontal Lobe, Superior Frontal Gyrus | 8 |
|  | 2 34 51 | 3.31 |  | <0.001 | Right Cerebrum, Frontal Lobe, Superior Frontal Gyrus | 8 |
| **LH associations with GMV among MHT non-users** |  |  |  |  |  |  |
| 16 | 35 33 24 | 3.70 | 0.010 | <0.001 | Right Cerebrum, Frontal Lobe, Middle Frontal Gyrus | 9 |
| 44 | 12 47 -21 | 3.55 | 0.003 | <0.001 | Right Cerebrum, Frontal Lobe, Orbital Gyrus | 11 |
|  | 14 39 -22 | 3.46 |  | <0.001 | Right Cerebrum, Frontal Lobe, Orbital Gyrus | 11 |
| 28 | 61 -23 -21 | 3.51 | 0.006 | <0.001 | Right Cerebrum, Temporal Lobe, Inferior Temporal Gyrus | 20 |

**P*<0.05 cluster-level corrected for Family-Type Wise Error (FWE) within the search volume, adjusting by total intracranial volume. Analyses were conducted using the subtraction analysis approach described in the methods section, where all voxel-wise regional contributions of age and menopause status were excluded from analysis using the explicit masking approach implemented in SPM12.
