# Supplementary material for: Elevated gonadotropin levels are associated with increased biomarker risk of Alzheimer's disease in midlife women
Source: Front Dement. 2023 Nov 23;2:1303256. doi: 10.3389/frdem.2023.1303256 (PMC11108587; doi:10.3389/frdem.2023.1303256)
Supplement: Supplementary file 4 [file Table_4.DOCX]

### Supplementary Table 4. Associations of FSH and LH levels with hippocampal volume 

|  |  | **Overall** |  |  | **Postmenopause** |  |  | **Perimenopause** |  |  |
| --- | --- | --- | --- | --- | --- | --- | --- | --- | --- | --- |
|  | Model | Coeff. | SE | *P* | Coeff. | SE | *P* | Coeff. | SE | *P* |
| FSH | 1 | -0.107 | 0.083 | *0.077* | -0.153 | 0.114 | *0.098* | -0.093 | 0.132 | 0.235 |
|  | 2 | -0.118 | 0.081 | *0.058* | -0.143 | 0.116 | 0.119 | -0.139 | 0.137 | 0.155 |
| LH | 1 | -0.034 | 0.083 | 0.180 | -0.040 | 0.113 | 0.367 | -0.014 | 0.150 | 0.456 |
|  | 2 | -0.047 | 0.082 | 0.266 | -0.036 | 0.119 | 0.382 | -0.044 | 0.157 | 0.375 |

Values are coefficients, standard errors (SE).

Borderline significant *P* values are in italics. Model 1: adjustment by age and menopause status; Model 2: further multivariable adjustment. All analyses are adjusted by total intracranial volume.
