# Supplementary material for: Elevated gonadotropin levels are associated with increased biomarker risk of Alzheimer's disease in midlife women
Source: Front Dement. 2023 Nov 23;2:1303256. doi: 10.3389/frdem.2023.1303256 (PMC11108587; doi:10.3389/frdem.2023.1303256)
Supplement: Supplementary file 5 [file Table_5.DOCX]

## e-Table 5. Associations of estradiol levels with MRI gray matter volume in AD-regions 

| Cluster extent | Coordinates x, y, z | Z | P_FWE_ cluster* | P voxel | Anatomical Region | Brodmann Area |
| --- | --- | --- | --- | --- | --- | --- |
| **Overall** |  |  |  |  |  |  |
| 236 | 12 47 -21 | 3.82 | <0.001 | <0.001 | Right Cerebrum, Frontal Lobe, Orbital Gyrus | 11 |
|  | -2 32 -21 | 3.78 |  | <0.001 | Left Cerebrum, Frontal Lobe, Rectal Gyrus | 11 |
|  | 12 30 -21 | 3.66 |  | <0.001 | Right Cerebrum, Frontal Lobe, Rectal Gyrus | 11 |
| 23 | 10 42 -10 | 3.76 | 0.015 | <0.001 | Right Cerebrum, Frontal Lobe, Medial Frontal Gyrus | 10 |
| 20 | -39 -50 -8 | 3.32 | 0.018 | <0.001 | Left Cerebrum, Temporal Lobe, Fusiform Gyrus | 37 |
| **MHT non-users** |  |  |  |  |  |  |
| 45 | 12 47 -21 | 3.80 | 0.006 | 0.000 | Right Cerebrum, Frontal Lobe, Orbital Gyrus | 11 |
| 37 | -39 -44 -7 | 3.74 | 0.008 | 0.000 | Left Cerebrum, Limbic Lobe, Parahippocampal Gyrus | 19 |
| 33 | -27 44 26 | 3.68 | 0.009 | 0.000 | Left Cerebrum, Frontal Lobe, Middle Frontal Gyrus | 10 |
| 77 | 0 37 -22 | 3.66 | 0.002 | 0.000 | Left Cerebrum, Frontal Lobe, Rectal Gyrus | 11 |
|  | -2 21 -20 | 3.58 |  | 0.000 | Left Cerebrum, Frontal Lobe, Rectal Gyrus | 11 |
| 21 | 33 34 36 | 3.61 | 0.015 | 0.000 | Right Cerebrum, Frontal Lobe, Middle Frontal Gyrus | 9 |

**P*<0.05 cluster-level corrected for Family-Type Wise Error (FWE) within the search volume, adjusting by total intracranial volume. Analyses were conducted using the subtraction analysis approach described in the methods section, where all voxel-wise regional contributions of age and menopause status were excluded from analysis using the explicit masking approach implemented in SPM12.
