# Supplementary material for: Elevated gonadotropin levels are associated with increased biomarker risk of Alzheimer's disease in midlife women
Source: Front Dement. 2023 Nov 23;2:1303256. doi: 10.3389/frdem.2023.1303256 (PMC11108587; doi:10.3389/frdem.2023.1303256)
Supplement: Supplementary file 6 [file Table_6.DOCX]

## e-Table 6. Voxel-based associations of FSH and LH with MRI gray matter volume excluding regional effects of estradiol 

| Cluster extent | Coordinates x, y, z | Z | P_FWE_ cluster* | P voxel | Anatomical Region | Brodmann Area |
| --- | --- | --- | --- | --- | --- | --- |
| **Overall FSH associations with GMV** |  |  |  |  |  |  |
| 67 | 14 32 -20 | 4.21 | 0.002 | <0.001 | Right Cerebrum, Frontal Lobe, Inferior Frontal Gyrus | 11 |
|  | 10 49 -20 | 3.91 |  | <0.001 | Right Cerebrum, Frontal Lobe, Orbital Gyrus | 11 |
|  | 12 57 -18 | 3.48 |  | <0.001 | Right Cerebrum, Frontal Lobe, Superior Frontal Gyrus | 11 |
| 17 | -10 52 27 | 3.78 | 0.013 | <0.001 | Left Cerebrum, Frontal Lobe, Superior Frontal Gyrus | 9 |
| 23 | 35 33 26 | 3.61 | 0.010 | <0.001 | Right Cerebrum, Frontal Lobe, Middle Frontal Gyrus | 9 |
| 52 | 47 25 -12 | 3.47 | 0.003 | <0.001 | Right Cerebrum, Frontal Lobe, Inferior Frontal Gyrus | 47 |
|  | 55 33 -10 | 3.42 |  | <0.001 | Right Cerebrum, Frontal Lobe, Inferior Frontal Gyrus | 47 |
| **Overall LH associations with GMV** |  |  |  |  |  |  |
| 22 | 12 45 13 | 3.69 | 0.003 | <0.001 | Right Cerebrum, Frontal Lobe, Medial Frontal Gyrus | 10 |
| 36 | 10 35 46 | 3.37 | 0.002 | <0.001 | Right Cerebrum, Frontal Lobe, Superior Frontal Gyrus | 8 |
| **FSH associations with GMV among MHT non-users** |  |  |  |  |  |  |
| 112 | 14 30 -21 | 4.28 | 0.001 | <0.001 | Right Cerebrum, Frontal Lobe, Inferior Frontal Gyrus | 11 |
|  | 10 49 -20 | 3.88 |  | <0.001 | Right Cerebrum, Frontal Lobe, Orbital Gyrus | 11 |
| 38 | 35 33 24 | 4.00 | 0.009 | <0.001 | Right Cerebrum, Frontal Lobe, Middle Frontal Gyrus | 9 |
| 63 | -8 -69 51 | 3.73 | 0.004 | <0.001 | Left Cerebrum, Parietal Lobe, Precuneus | 7 |
| 19 | 55 33 -10 | 3.30 | 0.021 | <0.001 | Right Cerebrum, Frontal Lobe, Inferior Frontal Gyrus | 47 |
| **LH associations with GMV among MHT non-users** |  |  |  |  |  |  |
| 16 | 35 33 24 | 3.70 | 0.007 | <0.001 | Right Cerebrum, Frontal Lobe, Middle Frontal Gyrus | 9 |

**P*<0.05 cluster-level corrected for Family-Type Wise Error (FWE) within the search volume, adjusting by total intracranial volume. Analyses were conducted using the subtraction analysis approach described in the methods section, where all voxel-wise regional contributions of age and menopause status were excluded from analysis using the explicit masking approach implemented in SPM12.
