# Supplementary material for: Elevated gonadotropin levels are associated with increased biomarker risk of Alzheimer's disease in midlife women
Source: Front Dement. 2023 Nov 23;2:1303256. doi: 10.3389/frdem.2023.1303256 (PMC11108587; doi:10.3389/frdem.2023.1303256)
Supplement: Supplementary file 7 [file Table_7.DOCX]

## e-Table 7. Associations between FSH, LH and cognitive scores

|  | **FSH** |  |  | **LH** |  |  |
| --- | --- | --- | --- | --- | --- | --- |
|  | Coeff. | Std. Err. | *P* | Coeff. | Std. Err. | *P* |
| **Overall** |  |  |  |  |  |  |
| global cognition | -0.061 | 0.057 | 0.426 | -0.021 | 0.057 | 0.781 |
| memory | -0.057 | 0.052 | 0.448 | -0.037 | 0.052 | 0.617 |
| **Post-menopause** |  |  |  |  |  |  |
| global cognition | -0.163 | 0.125 | 0.199 | 0.057 | 0.122 | 0.630 |
| memory | -0.144 | 0.113 | 0.207 | 0.043 | 0.108 | 0.711 |
| **Peri-menopause** |  |  |  |  |  |  |
| global cognition | -0.063 | 0.115 | 0.628 | -0.083 | 0.101 | 0.524 |
| memory | -0.068 | 0.105 | 0.518 | -0.108 | 0.093 | 0.396 |
| **Pre-menopause** |  |  |  |  |  |  |
| global cognition | -0.033 | 0.193 | 0.840 | -0.143 | 0.202 | 0.380 |
| memory | 0.082 | 0.187 | 0.612 | -0.060 | 0.196 | 0.710 |

Values are coefficients, standard errors (SE). Results are adjusted by age and education.
